# Microbial diversity and biogeochemical interactions in the seismically active and CO_2_- rich Eger Rift ecosystem

**DOI:** 10.1186/s40793-024-00651-9

**Published:** 2024-12-25

**Authors:** Daniel Lipus, Zeyu Jia, Megan Sondermann, Robert Bussert, Alexander Bartholomäus, Sizhong Yang, Dirk Wagner, Jens Kallmeyer

**Affiliations:** 1 GFZ German Research Centre for Geosciences, Section Geomicrobiology, Potsdam, Germany; 2 Department of Biological and Chemical Sciences, College of Life Sciences, Thomas Jefferson University, Philadelphia, Pennsylvania USA; 3 Section Applied Geochemistry, Institute of Applied Geosciences, Technische Universität Berlin, Berlin, Germany; 4 University of Potsdam, Institute of Geosciences, Potsdam, Germany

## Abstract

The Eger Rift subsurface is characterized by frequent seismic activity and consistently high CO_2_ concentrations, making it a unique deep biosphere ecosystem and a suitable site to study the interactions between volcanism, tectonics, and microbiological activity. Pulses of geogenic H_2_ during earthquakes may provide substrates for methanogenic and chemolithoautotrophic processes, but very little is currently known about the role of subsurface microorganisms and their cellular processes in this type of environment. To assess the impact of geologic activity on microbial life, we analyzed the geological, geochemical, and microbiological composition of rock and sediment samples from a 238 m deep drill core, running across six lithostratigraphic zones. We evaluated the diversity and distribution of bacterial and archaeal communities. Our investigation revealed a distinct low-biomass community, with a surprisingly diverse archaeal population, providing strong support that methanogenic archaea reside in the Eger subsurface. Geochemical analysis demonstrated that ion concentrations (mostly sodium and sulfate) were highest in sediments from 50 to 100 m depth and in weathered rock below 200 m, indicating an elevated potential for ion solution in these areas. Microbial communities were dominated by common soil and water bacteria. Together with the occurrence of freshwater cyanobacteria at specific depths, these observations emphasize the heterogenous character of the sediments and are indicators for vertical groundwater movement across the Eger Rift subsurface. Our investigations also found evidence for anaerobic, autotrophic, and acidophilic communities in Eger Rift sediments, as sulfur-cycling taxa like *Thiohalophilus* and *Desulfosporosinus* were specifically enriched at depths below 100 m. The detection of methanogenic, halophilic, and ammonia-oxidizing archaeal populations demonstrate that the unique features of the Eger Rift subsurface environment provide the foundation for diverse types of microbial life, including the microbial utilization of geologically derived CO_2_ and, when available, H_2_, as a primary energy source.

**Supplementary Information:**

The online version contains supplementary material available at 10.1186/s40793-024-00651-9.

## Introduction

The deep biosphere is one of the largest and most interesting ecosystems on Earth. Subsurface regions belonging to these systems are estimated to comprise up to one-third of the total global biomass and harbor a diverse array of geochemical settings and microbial habitats [1, 2]. While microbial life in marine subsurface sediments is relatively well constrained [3, 4], assessments of indigenous microbial communities and their contribution to biochemical cycles in deep terrestrial sediments and hard (i.e., igneous) rocks remain limited. As Earth’s terrestrial subsurface also represents an essential source of economically and societally important commodities, such as groundwater, minerals, metals and hydrocarbons, geochemical and microbial explorations into these systems are of great relevance. Although the often extreme conditions (no or little oxygen, no light, temperatures of more than 100° C, high pressures, limited carbon sources, and sometimes no water) are believed to push life to its limit and significantly decrease microbial turnover [5], previous studies have suggested Earth’s subsurface to harbor an unexpected phylogenetic diversity and accommodate a variety of unknown microbial populations, often referred to as microbial dark matter [6–9]. Explorations of metabolic processes in subsurface settings have asserted the importance of chemolithoautotrophic, and organotrophic lifestyles as well as methane cycling [10], emphasizing the dependence on geochemically and abiotically derived substrates such as H_2_ and CO_2_. Due to the scarcity of organic carbon, heterotrophic communities in deep continental subsurface settings are believed to rely on the production of fixed carbon through these autotrophic processes.

Subsurface environments characterized by high CO_2_ conditions have become research areas of particular scientific and industrial interest, as both artificial and natural reservoirs are being studied to evaluate the effects of geologic carbon sequestration, capture and storage (CSS), a process in which anthropogenic CO_2_ is injected into subsurface reservoirs for storage [6, 11–13]. In addition to influencing geochemical processes in the subsurface, such as lowering the pH and causing stronger weathering and mineral dissolution through elevated levels of CO_2_, CSS operations can significantly impact the indigenous microbial communities residing in these rocks. For example, saturated CO_2_ has been shown to interrupt microbial processes through cytoplasm acidification, while supercritical CO_2_ is becoming a standard sterilizing agent [14–17]. On the contrary, CO_2_ can also spark microbial growth by directly acting as a substrate for autotrophic growth or dissolving minerals, thus liberating nutrients or essential elements from the surrounding environment [12, 18–20]. Explorations of microbial distribution, diversity and metabolisms have accentuated the impact of CO_2_ in the terrestrial subsurface, showing that microbial populations in such settings are at least temporarily reduced [11, 12, 21] and the community composition shifted towards chemolithoautotrophic iron and sulfur oxidizing bacteria as well as methanogenic archaea. A limited number of metagenomic studies have evaluated the metabolic capabilities of CO_2_-adapted subsurface microbial communities. Freedman et al. [13] was able to recover *Thiobacillus*, *Gallionella*, and Hydrogenophales draft genomes from McElmo Dome aquifer samples recovered from 2600 m depth, reconstructing metabolic pathways such as carbon fixation via the Wood Ljungdahl pathway and Calvin Benson cycle, sulfur oxidation and partial nitrate reduction. Work by Emerson et al. [6] and Probst et al. [22] confirmed the relevance of these carbon fixation pathways, as similar genes were found to be enriched in 46 different genomes recovered from CO_2_-saturated Crystal Geyser subsurface fluids. Gulliver et al. [12] and Trias et al. [23] also reported the metabolic potential for carbon fixation and sulfur oxidation and detected an increased number of methanogenesis genes in subsurface aquifers subjected to CO_2_ injection. To date, analyses have provided strong evidence that microbial communities can at least temporarily adapt to high CO_2_ conditions [12, 13, 23], and only a few efforts have been made to evaluate microbial life in natural environments with continuous, long-term CO_2_ exposure [22]. Thus, additional work covering a wider range of naturally occurring high CO_2_ subsurface environments may provide further insights into the direct and indirect impacts of CO_2_ on microbial distribution and behavior and long-term development of such ecosystems.

The geodynamically active Eger Rift region in West Bohemia (Czech Republic) is part of the Počatky-Plesná Fault Zone (PPZ) and characterized by a rare combination of CO_2_-rich mantle degassing and regular seismic activity [24, 25]. Frequent earthquake swarms and high flow rates of mineral-rich fluids create a distinct lithological composition, making this region an excellent study site for evaluating microbial distribution, abundance, and processes under unusual deep subsurface conditions. Geogenic CO_2_ continuously migrates from active magma chambers at the crust-mantle boundary and from lithospheric mantle depths of about 65 km [26–28], resulting in CO_2_-rich conditions and the formation and accumulation of CO_2_ in aquifers [29]. At the surface, CO_2_-rich gas is discharged in the form of mofettes or mineral water springs [30–35]. In addition, periodically occurring earthquake swarms lead to the abiotic production of H_2_ in the Eger subsurface [27, 28]. The exceptional geo- and physicochemical conditions likely affect microbial development and activity, and may foster microbial processes through enhanced substrate generation [36], and potentially trigger a diverse range of rock-fluid interactions as part of geodynamic processes in the lithosphere [27, 35, 37] that can support microbial life.

However, in the Eger Rift subsurface high gas fluxes between 14 and 43 kg m^− 2^ d^− 1^with CO_2_ accounting for up to 99% of the soil gas content, can cause hypoxia and acidification of the soil, mobilization of metals and thereby may limit or even preclude microbial life [38–40]. In addition, previous work has suggested high CO_2_ concentrations can suppress heterotrophic growth thereby favor the emergence of other secondary metabolic traits [41].

Several efforts have been made to study microbial life and the microbial responses to geological processes in the Eger Rift region. A 2005 study by Bräuer et al. [28] described a short peak of elevated concentrations of H_2_ followed by a peak in biogenic methane in mineral spring waters after a seismic event, suggesting that abiotically derived H_2_ can provide the foundation for microbial life in the Eger Rift subsurface. In this proposed scenario the released H_2_, together with the abundantly available CO_2_, may trigger a dormant methanogenic community, resulting in the autotrophic production of methane, and thereby providing the basis for secondary, heterotrophic metabolic activity [28]. A recent study by Jia et al. [42] showed methanogenic archaea from Eger Rift sediments to become active under high H_2_/CO_2_ conditions. In addition, investigations of microbial composition and activity in mofette and mineral spring waters as well as surface sediments from the Cheb Basin have highlighted the role of acidophilic and methanogenic microbial processes in response to elevated levels of CO_2_ [29, 43, 44]. Several studies also reported on the importance of sulfur and iron oxidizing and CO_2_ fixating microbial communities in these microhabitats [11–13, 22, 41, 43]. While these explorations provided some insights into how these unique geochemical conditions may shape microbial life across the Eger subsurface, to this date only one study [29] has attempted to directly access and characterize microbial populations in Eger Rift subsurface sediments and rock formations. In 2016 a 108.5 m long core was recovered from the Hartoušov Mofette Field, followed by analyses of and its geochemistry and microbiology [29, 31]. Lithology analyses revealed Cenozoic sediments in the form of grey to brown and sandy to peaty mudstones, with lignite layers and root structures up to a depth of 90 m. Below 90 m core sections were characterized by weathered schists [31]. The recovery of CO_2_-rich sediments and the identification of a CO_2_-saturated saline aquifer around a depth of 80 m [31] further emphasized the significant role of CO_2_ in this ecosystem.

Microbial investigations targeted a 30 m section around the CO_2_-rich aquifer and resulted in the detection of a low biomass community, characterized by water and soil bacteria, specifically of the class Gammaproteobacteria [29]. Only minor signatures of microorganisms usually observed in acidic, high CO_2_ environments were detected, including low levels of methanogenic archaea and potentially autotrophic Comamonadaceae. The abundance and distribution of microorganism in the Eger Rift subsurface was linked to frequently changing groundwater levels, as pump test data from the borehole suggest the presence of major fluid ascending channels, which indicate reoccurring vertical groundwater movement. Even though this earlier drilling endeavor offered a valuable, first look into the microbial distribution and composition of Eger Rift subsurface sediments, the microbiological analyses solely focused on the area around the CO_2_-rich aquifer, effectively only providing a limited glimpse of what microbial life in this subsurface ecosystem may look like. To further extend the current understanding of the terrestrial biosphere in the Eger Rift and specifically evaluate the role of geologically derived compounds or substrates contributing to the development of microbial populations, additional efforts targeting broader regions of the Eger subsurface are needed.

In an effort to close this knowledge gap and advance explorations of microbial life in the Eger Rift subsurface, we evaluated drill core samples from a recent drilling campaign in the Hartoušov Mofette Field, which reached a depth of 238 m. The main objective of this campaign was to provide a deeper and more comprehensive description of the microbiological composition, and to specifically evaluate the diversity and distribution of archaea, as especially methanogenic Euryarchaeota may have the metabolic capability to utilize geogenic H_2_ in the presence of CO_2_.

We hypothesized that the geophysical conditions, including the consistent CO_2_ degassing, shape microbial life in the Eger Rift subsurface, resulting low-diversity ecosystem, characterized by few abundant microbial taxa that have the metabolic capability to utilize CO_2_. We specifically expected to detect microbial communities contributing to the oxidation of inorganic compounds, including sulfur-oxidizing and CO_2_ fixing microorganisms, acidophilic and potentially halophilic taxa, and methanogenic archaea. Based on previous observations, we also hypothesized that both the geochemical and microbiological composition of the recovered sediment and rock samples are affected by vertical groundwater movement and the existence of the described CO_2_-rich aquifer as well as similar structures, which may exist in deeper, previously uncharacterized regions of the Eger subsurface.

Using qPCR, 16 S rRNA sequencing and fluorescence microscopy we were able to assess microbial abundance and composition patterns across 26 samples, covering depths between 17 m and 230 m. Analysis of water-soluble cation and anion concentrations across the core helped us to identify areas of increased ion dissolution, potential groundwater movement and CO_2_ accumulation, while microbial explorations provided novel insights into a microbial community characterized by an unexpectedly high archaeal diversity. Findings from this study provide additional insights into the microbial community structure found across the Eger Rift subsurface and advance the overall understanding of natural high CO_2_ subsurface ecosystems. Together with the data from previous Eger Rift explorations, our observations also provide the foundation for future efforts studying the interactions between volcanism, tectonics and microbiological activity in terrestrial subsurface environments with the goal to elucidate the impact of geological processes on the deep biosphere.

## Materials and methods

### Site description

**Fig. 1:**
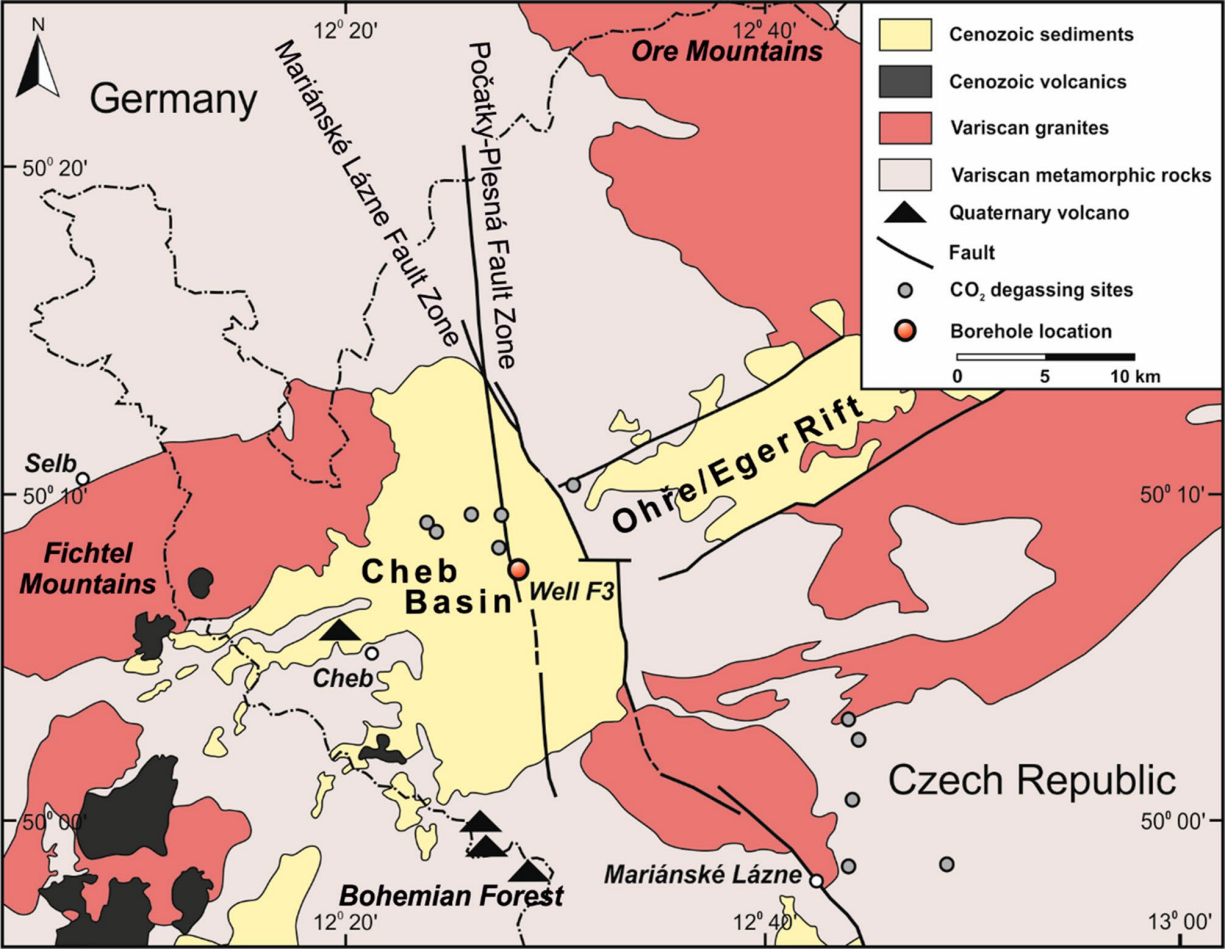
Geological map showing the location of the F3 borehole in the Cheb Basin in the western part of the Eger Rift (Czech Republic)

Drilling of the F3 borehole was conducted in August 2019 at the Hartoušov Mofette field (HMF) in the Cheb Basin in the western part of the Eger Rift (Fig. 1). This part of the Cheb Basin and the HMF in particular have been extensively described in previous studies [31, 44–46]. The HMF is known for its unique patterns of CO_2_ degassing, with the most heavy degassing observed in the central and northern parts with emission rates of up 43 kg m^− 2^ d^− 1^ [24, 47]. The drilling position was in close proximity to that of the previous two drill sites (F1 and F2). The F1 drilling was conducted in 2007 [30, 48] and reached a depth of approximately 28 m below ground into a CO_2_-saturated aquifer. The F2 borehole [48] was drilled in 2016 to a depth of ∼108 m. The F2 drilling campaign was conducted to evaluate whether the increased fluid and substrate flow can accelerate microbial life in active fault zones and CO_2_ conduits [29, 31].

### Drilling and coring

The 2019 drilling campaign started on August 9th, 2019. First a bunker down to a depth of 10 m was excavated and cemented. The upper 42 m were then drilled using PQ-sized equipment (122 mm hole diameter, 85 mm core diameter), followed by the installation of steel casing. In this section the cores were retrieved in PVC liners of 1 m length and only water without any additives was used as the drill mud. The uppermost recovered sediments were mostly unconsolidated sand and gravel. Sample collection for geochemical and microbiological analysis started at a depth of 17 m downwards. From 40 m on, drilling proceeded using HQ tools with a core diameter of 63.5 mm (96 mm hole). Core sections were retrieved in 3 m aluminum split liners. Drilling continued down to a depth of 238 m, with a total core recovery of 163.15 m (82.3% recovery rate). For deeper drilling, the drill mud consisted of bentonite and tap water. To account for possible contamination of core material through infiltration of drill mud into the core, drilling was carried out under strict contamination control using a fluorescent microsphere tracer, as reported previously [49, 50]. Tracer was added periodically to the drill mud and monitored to maintain a 1:1000 dilution and a concentration of approximately 10^9^ particles per mL drill fluid. Drill mud samples were collected after core recovery, checked on-site, and stored for downstream analysis (Table S1).

### On-site core subsampling and processing

After being brought up, core sections were immediately transferred to the mobile geomicrobiology laboratory container of the GFZ Potsdam (BugLab) located in the vicinity of the drill rig to allow core subsampling and sample processing under optimal conditions. Whole round cores (WRC) of 10 cm length were cut from the recovered cores. For downstream geochemical and culturing experiments, the out layer was removed from the WRCs and they were immediately placed in CO_2_-flushed gastight bags to preserve native conditions. The samples were stored at 4^o^C until analysis. Whole round cores intended for molecular analysis had the outer, drill mud-contaminated rim removed on site (~ 1 cm), placed in gastight bags, and immediately frozen in liquid nitrogen until arrival at GFZ Potsdam, where they were transferred to a -80^o^C freezer.

Remaining core sections were logged, cut into 1 m pieces, labeled, stored in wooden core boxes and transported to the “Deutsches Bohrkernlager für kontinentale Forschungsbohrung” of the German Federal Institute for Geosciences and Natural Resources in Berlin Spandau for core description and long-term storage.

### Sample processing for genomic work

Frozen core sections were slightly thawed and processed in a sterile laminar flow clean bench (Thermo Scientific, Waltham, USA). To avoid drill mud contaminated material, samples were collected from the inner section of the WRC. In addition, WRC subsamples were taken for assessment of contamination. Up to 15 g of the collected inner core material was stored in 50 mL centrifuge tubes and kept frozen at -80^o^C until DNA extraction. As different DNA extraction approaches were employed, up to 1 g of core material was stored in 2 mL screw-cap tubes at -80^o^C.

### Ion leaching

The low water content of the core material did not allow collection of a sufficient amount of pore water via squeezing. To assess the ionic composition of the aqueous phase, samples were leached [51]. Briefly, 5 g of dried sample were suspended in 25 ml deionized water and agitated on a horizontal shaker for 90 min at 175 rpm. The samples were then centrifuged for 10 min at 175 rpm and the supernatant collected. The supernatant was filtered through a 0.45 μm filter and stored at 4^o^C. Duplicate samples from each core section were processed.

### Ion Chromatography

The overall conductivity of the collected leachate was assessed using a TDS meter (WTW Multi 3420). Anion and cation compositions of the leached pore water were measured using ion chromatography (IC) (Sykam Chromatography, Fürstenfeldbruck, Germany). For anions, the system consisted of an S5250 sample injector, an S150 IC-module, an anion separation column, and an S3115 conductivity detector. The eluent was 6 mM Na_2_CO_3_ and 9 mM NaSCN, the flow rate was set to 1 mL min^− 1^, oven temperature was 50^o^C. Multi element standards from Sykam and Carl Roth were used. All samples were diluted 10X and measured in technical triplicates. The reproducibility was within 1% intrinsic error for both cation and anion measurements.

The cation chromatography system consisted of an S5300 sample injector, ReproSil Cat separation column, and S3115 conductivity detector. The flow rate was set to 1.2 mL min ^− 1^, the eluent was 5 mM H_2_SO_4_ and the column temperature was set to 45^o^C. A 10-times diluted cation multi IC standard (Carl Roth) was used. Samples were measured undiluted as technical triplicates. IC chromatographs were analyzed using Chromstar7 software.

### TOC and TN measurements

Dried soil samples were grinded up into a fine powder with a stainless-steel mill (Retsch, Germany) for subsequent analysis of total organic carbon (TOC) and total nitrogen (TN) using an elemental analyser (Flash EA 2000, ThermoFisher Scientific). Briefly, 6 to 10 milligrams of pulverized soil was filled in Ag-capsules (EuroVector) and treated with 3% and 20% HCl to remove carbonates. The acid treatment with 3% HCl was repeated until no foam formation was observed. The treatment with 20% HCl was performed as the final step. Acid-treated soil samples were dried at 75 °C for 3 h and folded before the analysis. All samples were measured in at least a duplicate. Calibration was performed using commercial reference soil samples (EuroVector).

### Cell counts

For cell counts, ~ 3 g of drill core sediment were placed in a 50 ml Falcon tube and mixed with 2x volumes of 4% filter-sterilized 2% (v/v) formaldehyde solution.

Cell extraction was then carried out according to Kallmeyer et al. 2008 [52] with some modifications. For each fixed sample, 100–600 µL of homogenized slurry was mixed with 200 µL 2.5% (w/v) NaCl solution, 50 µL detergent mix (100 mM Na2EDTA•2H2O, 100 mM Na4P2O7•10H2O, 1% (v/v) Tween-80, autoclaved and filter-sterilized after cooling) and 50 µL methanol. The mixture was vortexed for 30 min. 500 µL of filter-sterilized 50% (w/v) Nycodenz (Axis-Shield) solution was added to the bottom of the vial using a syringe needle (18-gauge). After centrifugation at 3000 ×g for 10 min, 800 µL of supernatant was carefully collected and transferred to another tube. The remaining Nycodenz solution above the pellet was discarded, and 300 µL of 2.5% (w/v) NaCl solution, 50 µL detergent mix and 50 µL methanol were added to the pellet. The mixture was sonicated in an ice-water bath for 4 × 10 s, with 20 s between cycles at 25% power (BANDELIN SONOPULS, with ultrasonic probe MS73). Then a second time of Nycodenz density centrifugation was performed on the ultrasonicated mixture. The collected 800 µL supernatant was pooled with that from the first round and preserved for cell counting. To further dissolve the remaining clay-like particles in the supernatant, a 1% (v/v) HF solution was added at a volume ratio of 1:1. During HF treatment, the mixture was placed in an ultrasonic bath for 15 min. After a total treatment time of 30 min, 5 mL of 0.5 M EDTA solution was added to avoid excessive damage to cells due to HF. The total liquid was passed through a 0.2 μm-pore-size filter membrane (GTBP, Millipore). 2 mL of detergent mix was added before filtration to obtain a more even distribution of the sample on the surface of the filter membrane.

Cells on the filter were stained and mounted on a glass slide with SYBR solution (300 µL VECTASHIELD mounting medium H-1000, 300 µL glycerol, 100 µL 1% (w/v) p-phenylenediamine, 10 µL 10000X SYBR Green I stock solution, 290 µL H_2_O). Cells were counted with an epi-fluorescence microscope (DM2000, Leica) equipped with an oil-immersion objective (HCX PL APO 100×/1.40, Leica) and light filter sets of either Leica I3 or L5 [excitation filter of 470/40 nm and 480/40 nm (band-pass filter, center wavelength/bandwidth, same below) respectively, and a suppression filter of 515 nm (long-pass filter, initiate wavelength) and 527/30 nm, respectively]. For each slide, at least 200 cells were counted unless 200 fields of view were screened.

### DNA extraction, clean up, and concentration

Based on the experience of Liu et al. [29, 31], we expected that obtaining a sufficient amount of high-quality DNA from the recovered core sections would be one of the major challenges of our study. To ensure optimal DNA recovery, we employed three different DNA extraction methods.

First, DNA was extracted from up to 1 g of sediment using a modified version of the Qiagen DNeasy PowerSoil kit (Qiagen, Venlo, Netherlands), as described previously [53, 54]. The major changes from the manufacturer’s protocol were the addition of 10 mg/l lysozyme followed by a 1-hour incubation step at 50^o^C prior to bead beating. We also added three different types of zirconia beads instead of the kit-supplied beads and used a 45 s bead beating step at 5 m/s instead of the protocol standard vortexing. To ensure maximum recovery, the sample was extracted twice; after centrifugation and removal of lysate post bead beating, another volume of extraction buffer was added, and the bead-beating and the centrifugation step were repeated. The remaining precipitation and DNA binding steps were carried out as described in the manufacturer’s protocol. Negative controls were included in every extraction round.

DNA was also extracted using a modified Phenol-Chloroform-based method after Nercessian et al. [55]. Similar to the kit approach, 0.5–1.0 g of sediment were treated with lysozyme and 800 µl of an EDTA (0.5 M) and phosphate-based extraction buffer (0.12 M) at 50^o^C for one hour followed by the addition of two sizes (0.1 mm and 0.7 mm) of zirconia beads and one size (3–4 mm) of glass beads. An equal volume of chloroform-isoamylalcohol and 10% SDS was added and the samples underwent bead beating for 45 s at 5 m/sec. Samples were then centrifuged at 16,000 x g for 10 min at 4^o^C. The lysate (supernatant) was collected and the bead-beating step was repeated with another volume of extraction buffer. DNA was then precipitated by adding 0.5 volumes of isopropanol followed by incubation for one hour at room temperature. DNA was pelleted by centrifugation at 17,000 x g for one hour at 4^o^C. DNA pellets were washed with 70% ethanol and centrifuged at 17,000 x g for 10 min. DNA pellets were air-dried and eluted in 100 µl DNase free, PCR grade H_2_0. To achieve sufficient DNA, extractions were performed in quadruplicates for each sample. Eluted DNA was then pooled and concentrated by precipitation with 100% Ethanol and 0.2 M NaCl. Negative controls were included in each extraction run.

The third method used for DNA extraction in this study was based on a protocol by Rohland et al. [56]. First, core material (5.0–8.0 g) was added to a 50 ml falcon tube. Then 20 ml of an EDTA-based extraction buffer were added (for 25 ml: 22.5 ml 0.5 M EDTA, 1.86 ml sterile PCR grade water, 12.5 µl Tween 20, 62.5 µl mg/ml Proteinase K). Samples were then incubated overnight (15–18 h) at 37^o^C in a horizontally shaking incubator (80 rpm). Post incubation, the supernatant was collected by centrifugation at 16,400 x g for 2 min. The supernatant was then mixed with 2 volumes of binding buffer (5 M guanidine hydrochloride, 40% (vol/vol) isopropanol and 0.05% (vol/vol) Tween 20). DNA was collected by running 5 ml of the mixture over a Zymo silica spin column by centrifugation in a swing-out rotor at 500 x g for 5 min. The flow-through was discarded, and the step repeated until the entire mixture was run through the spin column. The spin column was transferred to a small 1.5 ml low bind tube and the DNA on the spin column was washed using an ethanol-based wash buffer (50% EtOH, 125 mM NaCl, 1mM EDTA). DNA was eluted in 80 µl PCR-grade H_2_0.

To limit bias through the applied DNA extraction protocol, DNA from all three methods was pooled and cleaned using AMPure XP beads (Beckman Coulter, Pasadena, CA, USA). DNA concentrations were assessed using Qubit (Life Technologies, Carlsbad, CA, USA) and an Agilent tape station (Agilent, Santa Clara, CA, USA).

We also attempted to extract DNA for metagenomic library preparation, however we were not able to obtain sufficient quantity or quality. We attempted to generate metagenomic libraries from these DNA extracts both in-house and through external providers, but were not able to obtain adequate metagenomic libraries.

### Polymerase chain reaction (PCR) and Illumina sequencing

The V4 region of the 16 S rRNA gene was amplified using universal 515 F and 806R barcoded primers [57, 58], as described previously [29]. PCR reactions were run as quintuples for each sample for 30 cycles (30s at 95^o^C, 45s at 56^o^C, 60s at 72^o^C) to minimize the introduction of contamination. Extraction and template (PCR) controls were included in each PCR run using their own set of barcoded primers. *E. coli* and microbial mock community DNA were included as positive controls. PCR products from the same samples were then pooled and cleaned using AMPure XP beads (Beckman Coulter, Pasadena, CA, USA). Concentration of PCR products was assessed using Qubit technology (Life Technologies, Carlsbad, CA, USA) and pooled in equimolar amounts. The pooled DNA library was concentrated with an Eppendorf Concentrator plus (Eppendorf AG, Hamburg, Germany) and sequenced on an Illumina Miseq Sequencer by a Eurofins Scientific SE (Munich, Germany).

### Quantitative PCR

Abundance of microorganisms was also assessed by quantitative PCR targeting the bacterial 16 S rRNA gene using a BIO-RAD CFX Connect Real-Time System (Bio-Rad Laboratories, California, USA) with primer Eub341-F/Eub534-R and SensiFAST SYBR mix. Quantitative PCR conditions were: 95^o^C for 3 min, followed by 35 cycles for 3 s at 95^o^C, 20 s annealing at 60^o^C, 30 s at 72^o^C, and 3 s plate read at 80^o^C. DNA for standard curves was cloned from *E. coli* (bacterial 16 S rRNA gene), as previously described (Wojcik et al., 2018). The analysis of the data was performed using the CFX Manager software of Bio-Rad.

### Bioinformatic and statistical analysis

Sequencing data was processed using the QIIME2 version 2019.10 [59]. Dual indexed sequencing reads were demultiplexed using CutAdapt [60]. DADA2 [61] was used to filter, denoise, and remove chimeras from the demultiplexed sequencing data. This included initial sequence truncations (250 bp forward reads, 200 bp reverse reads). Quality-filtered paired end reads were then merged. All final sequences had a standardized read orientation and a minimum length of 200 bp. A sequence table was created resulting in Amplicon Sequence Variants (ASVs). The classify-sklearn [62] command was used to classify representative sequences identified through DADA2 using a pre-trained Naive Bayes classifier trained on Silva taxonomy database (v132) and assign taxonomic units (ASVs, 99%) [63, 64]. Singletons and ASVs assigned to chloroplasts and mitochondria were removed from the ASV table. In addition, the resulting The pooled DNA library was concentrated with an Eppendorf Concentrator plus (Eppendorf AG, Hamburg, Germany) and sequenced on an Illumina Miseq Sequencer by a Eurofins Scientific SE table was manually screened and curated for contamination based on negative controls.

For illustration purposes taxonomic composition was summarized at the class and genus level. Class level taxonomy was visualized using the complex heatmap package in R [65]. The relative abundance of the most abundant 25 occurring ASVs was visualized on a bubbleplot using the phyloseq and ggplot2 packages in R [66–68].

The final curated ASV table was subsampled for a sequencing depth of 3,708 sequences (lowest sequence count) for alpha diversity analysis. Subsampling was conducted using the phyloseq package in R [66]. Microbial diversity was assessed by calculating observed ASVs, Shannon indices, and evenness indices using the estimate_richness command in phyloseq in R [66].

Inter sample diversity (beta-diversity) was determined by non-metric multidimensional scaling (NMDS) using Bray-Curtis dissimilarity distances in PAST4 [69]. The ASV table was Hellinger transformed for this analysis. Environmental parameters (here conductivity and ion concentrations) were z-score normalized fitted onto the ordination plot as vectors. Differences in microbial community structure among samples defined by environmental and physical parameters (depth, ionic strength) were assessed using analysis of similarity (ANOSIM) and permutational multivariate analysis of variance (PERMANOVA) calculations in Past4. Potential significant correlations between environmental parameters and biological data were examined and visualized using the cor function in R.

### Deposition of sequencing data

The Illumina MiSeq sequencing data of the generated 16 S rRNA libraries were deposited at the European Nucleotide Archive (ENA). The dataset can be accessed at ENA or the National Center for Biotechnology Information (NCBI) under the accession number #PRJEB55581.

## Results

### Drilling and sampling

Drilling reached a final depth of 238 m. Overall, 210.2 m of drill core were collected, resulting in a recovery rate of 88.3%. Core processing and sampling resulted in the collection of 55 samples, 28 for geochemical and 27 for biological analyses (Table S1). Two geochemical samples were compromised as the packaging broke during storage and transport, leading to sample oxidation. In addition, in two cases, no microbiological samples could be taken, because the drill core material was crumbly and visibly contaminated by drill mud. Drill mud samples were collected periodically, and eight depths were selected as sequencing controls (Table S1). On-site observations suggested that core sections below a depth of 50 m were frequently saturated with CO_2_, as the release of CO_2_ from the core was visible through bubbling and could be heard upon close examination.

### Core description

**Fig. 2:**
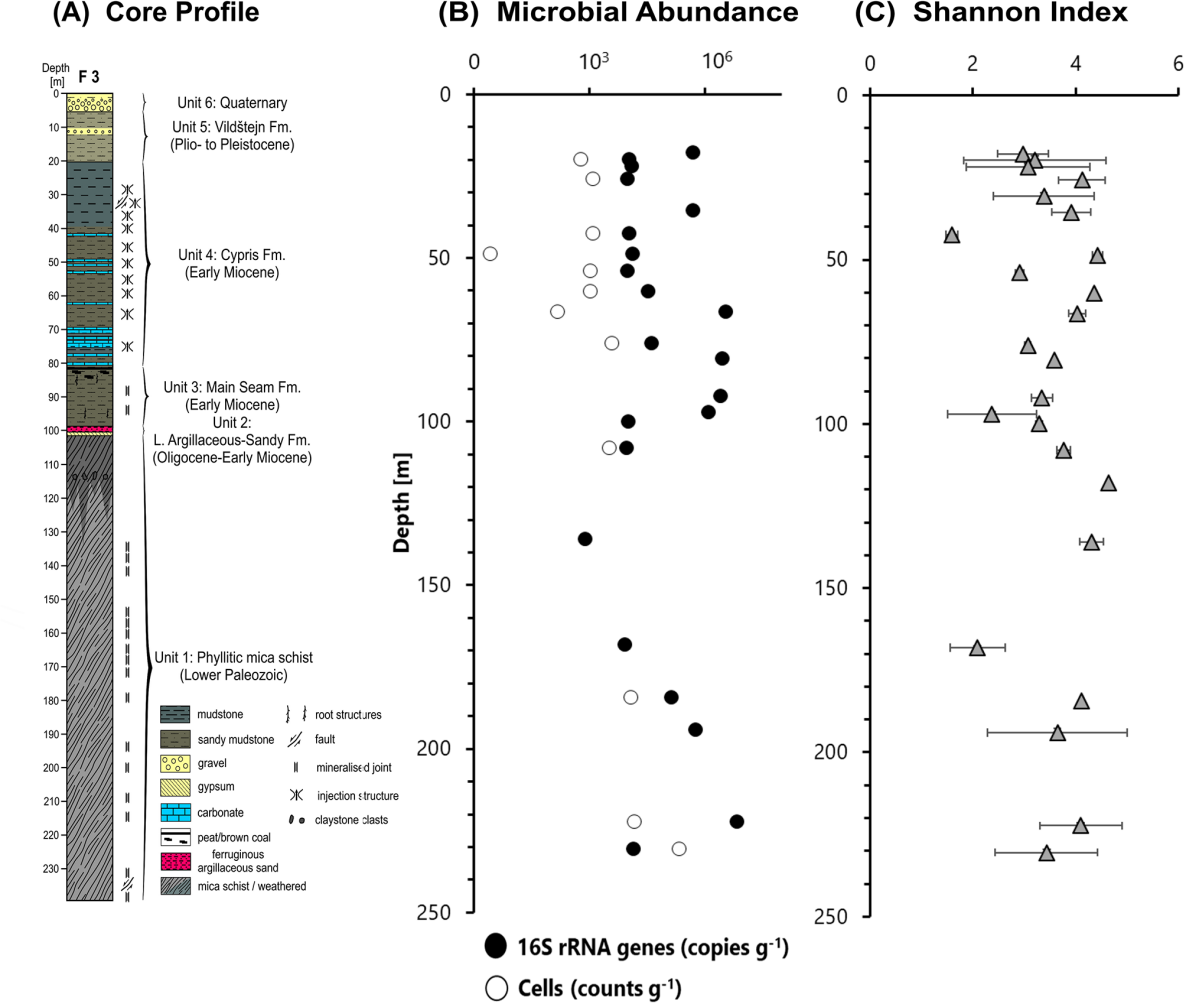
Stratigraphically and lithological description (**A**) of recovered Eger drill core (larger and detailed version of image in additional file, Figure S1), microbial abundance (**B**) measurements as determined by qPCR (16 S rRNA gene copies) and fluorescent microscopy (cell counts), and diversity measurements depicted as Shannon Index (**C**)

The F3 well core was divided into six lithostratigraphic units, as shown in Fig. 2 and Figure S1 (large image of core profile). The lowest unit, Unit 1, extends from 239.5 to about 100.0 core meters and consists of fine to medium-grained phyllitic mica schists primarily composed of quartz, feldspar and mica. The schists belong to the Lower Paleozoic Saxothuringian basement of the Cheb Basin [60]. Pyrite crystals, up to several millimetres in size, are scattered throughout the schists or concentrated along planes of schistosity and mineralized joints. At a depth of around 235 m, the presence of a fault zone is indicated by a fracture plane with a dark-brown clay gouge and a surrounding fractured zone with numerous pyrite crystals. In several core intervals, the mica schists are intersected by veins filled with pyrite, quartz or siderite. In irregularly distributed but upward increasingly frequent intervals, the mica schists lack clear foliation and are predominantly composed of quartz, kaolinite and siderite. Gypsum veins occur in the uppermost section of Unit 1.

The overlying Unit 2 may only be present in the well from around 100.0 m to 99.65 m, but a core loss of almost 1.3 m does not allow a definitive conclusion to be drawn as to its thickness. It is a reddish-coloured clayey-silty sandstone that probably belongs to the Lower Argillaceous-Sandy Fm [62]. It consists of quartz, kaolinite, siderite, hematite and muscovite, indicating intense chemical weathering during deposition.

Unit 3 spans from ~ 98.36 m to ~ 80.50 m and consists mainly of massive, gray to brown, sandy to peaty mudstones. This unit most likely represents the Early Miocene Main Seam Fm [62]. The mudstones are composed of kaolinite, siderite, quartz and anatase, with occasional greigite and crandallite. Additionally, several gypsum veins cross the mudstones.

Unit 4, ranging from about 80.5 to 20 m, most likely represents the Early Miocene Cypris Fm. This unit is mainly composed of gray to green laminated mudstones that consist primarily of clay minerals such as muscovite/illite, kaolinite, smectite and mixed layer minerals. Other minerals present include quartz, potassium feldspar, pyrite, greigite, zeolite, gypsum, and analcime. Sandy and partly peaty mudstones, as well as carbonate layers, occur particularly in the lower interval of the unit. Unit 4 represents deposits in a relatively deep lake. The sediments in this unit are frequently soft-sediment deformed, often in the form of sedimentary dikes surrounded by alteration zones. The deformations seem to be primarily injection structures that may have been caused by the ascent of CO_2_-rich deep crustal fluids.

Unit 5 is occurs between ~ 20 m and ~ 5.35 m depth. It consists mainly of green to gray sandy clay with intercalated gravel beds. This unit most likely represents lacustrine and alluvial sediments of the Pliocene to Pleistocene Vildštejn Formation [70].

The uppermost unit 6, extending from ~ 5.35 m to the surface, is primarily made up of clayey sand and gravel, forming fining-upward cycles a few decimeters thick. These sediments are almost certainly Quaternary channel and floodplain deposits of the nearby Plesná River.

### Geochemical composition

**Fig. 3:**
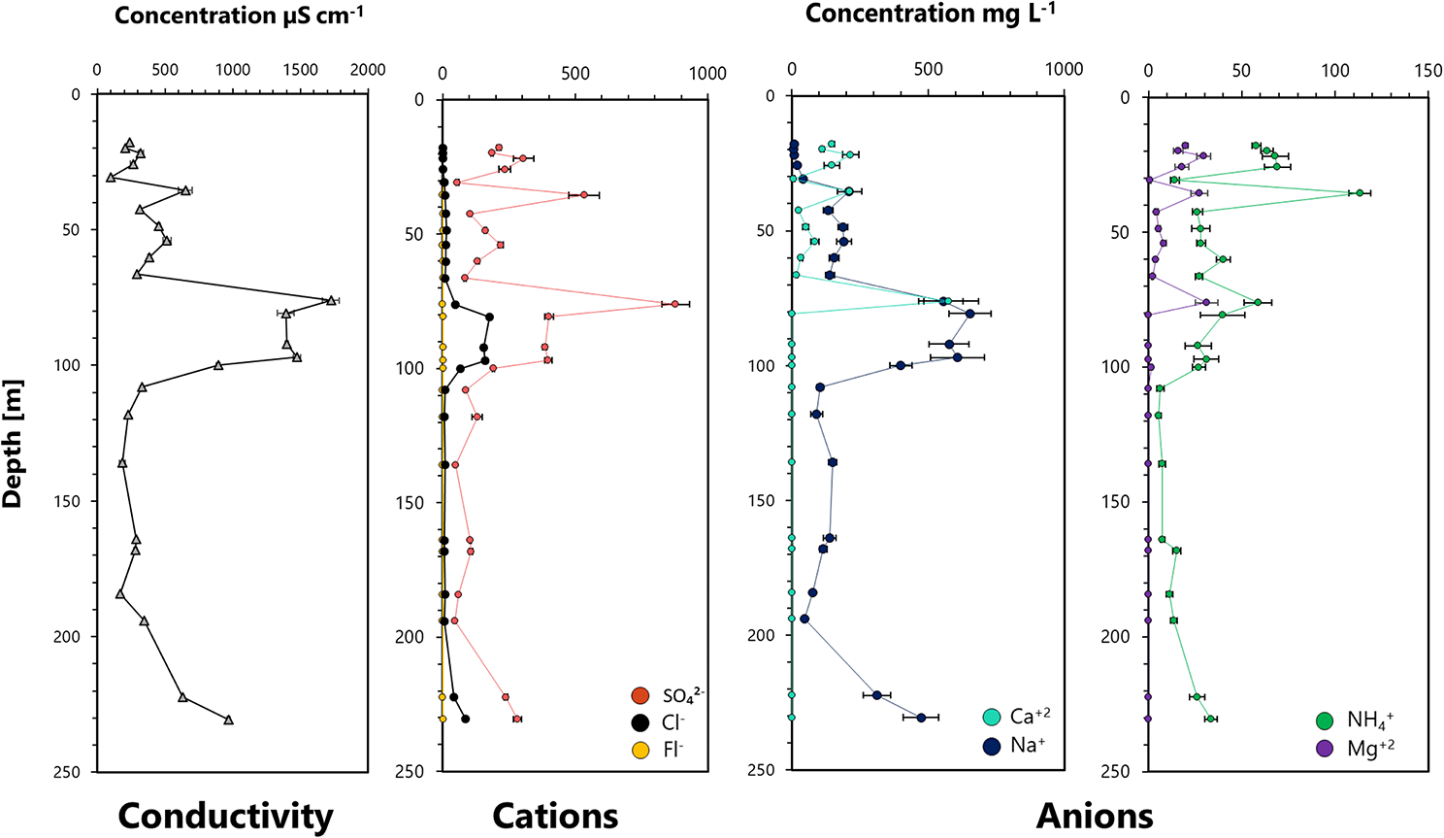
Geochemical composition, including overall conductivity, cation and anion concentrations across the recovered drill core profile

The overall conductivity of the leaching products ranged between 98 µS cm^− 1^ and 1726 µS cm^− 1^ (Fig. 3). Concentrations were relatively low across the upper 70 m and peaked between 75 and 100 m. Sediments from the intermediate section of the core (100–200 m) were characterized by a lower ionic content, while below a depth of 200 m, the conductivity increased again. To investigate the ionic distribution more closely, ion chromatography measurements of anions and cations were carried out.

Sulfate (up to 878.4 mg L^− 1^, 9.14 mM) and Sodium (up to 653.6 mg L^− 1^, 28.4 mM) were the most abundant ion species (Fig. 3, Table S2). Levels were highest in sediments from depths between 80 and 100 m, and increased again in rock samples from lower core sections. Chloride (up to 176.4 mg L^− 1^, 4.97 mM) and Ammonium (up to 113.3 mg L^− 1^, 6.28 mM) concentrations were lower overall, but followed the same pattern (Fig. 3, Table S2). Calcium and Magnesium were only detected in sediments from the upper 76 m. Concentrations were elevated in the uppermost sediments (215.6 mg L^−^1 Calcium (5.38 mM) and 29.6 mg L^− 1^ Magnesium (1.21.mM) at 22 m) and then peaked at 76 m (576.0 mg L^− 1^ Calcium (14.37 mM), 31.2 mg L^− 1^ Magnesium (1.28 mM). Fluoride was barely detectable and was highest in sediments from around 30 m (2.3 mg L^− 1^ (0.05 mM)).

Principal component analysis (PCA) suggested ion composition to be associated with formation and thus depth, as samples from the main seam formation and deep phyllitic mica schist were found to cluster together (Figure S2).

### Microbial abundance and diversity

To assess the overall microbial abundance, distribution, and diversity in Eger Rift sediments, we employed fluorescence microscopy, qPCR and 16 S rRNA sequencing. Despite the difficult sample material, it was possible to recover DNA and prepare sequencing libraries from 24 different drill core sediment samples covering depths between 17 and 230 m (Table S3).

DNA extractions and 16 S rRNA sequencing were performed in duplicate or triplicate for the majority of the samples (Table S3). Sequencing generated between 3,708 and 133,896 sequences (post trimming and contamination removal) per sample, with an average sequencing depth of 43,603 sequences (Table S3).

Quantitative PCR (qPCR) measurements confirmed the low biomass environment as measured values were just above the detection limit for most samples, ranging between 10^2^ and 10^6^ 16 S rRNA gene copies per gram of sediment extracted (Table S3, Fig. 2). The highest values were detected between 60 and 100 m and in sediments recovered from depths below 200 m.

Cell counts using fluorescence microscopy were used to obtain an additional assessment of the overall biomass in the recovered drill core sediments but proved to be challenging. Cells were counted in 13 samples and counts ranged between 10^1^ and 10^5^ cells g^− 1^ (Table S3, Fig. 2). Cell counts were highest in the deepest sediment below a depth of 185 m.

Overall microbial diversity was assessed by determining Shannon alpha diversity indices and assessing the number of observed ASVs per 3,708 sequences. Highest diversities were observed around 50 m (414 ASVs, Shannon 4.3) and 120 m (576 ASVs, Shannon 4.6), whereas the lowest number of ASVs and the lowest Shannon index were detected at 42 m and 168 m (Fig. 2). However, no significant trends or differences were identified across samples.

### Inter sample diversity

**Fig. 4:**
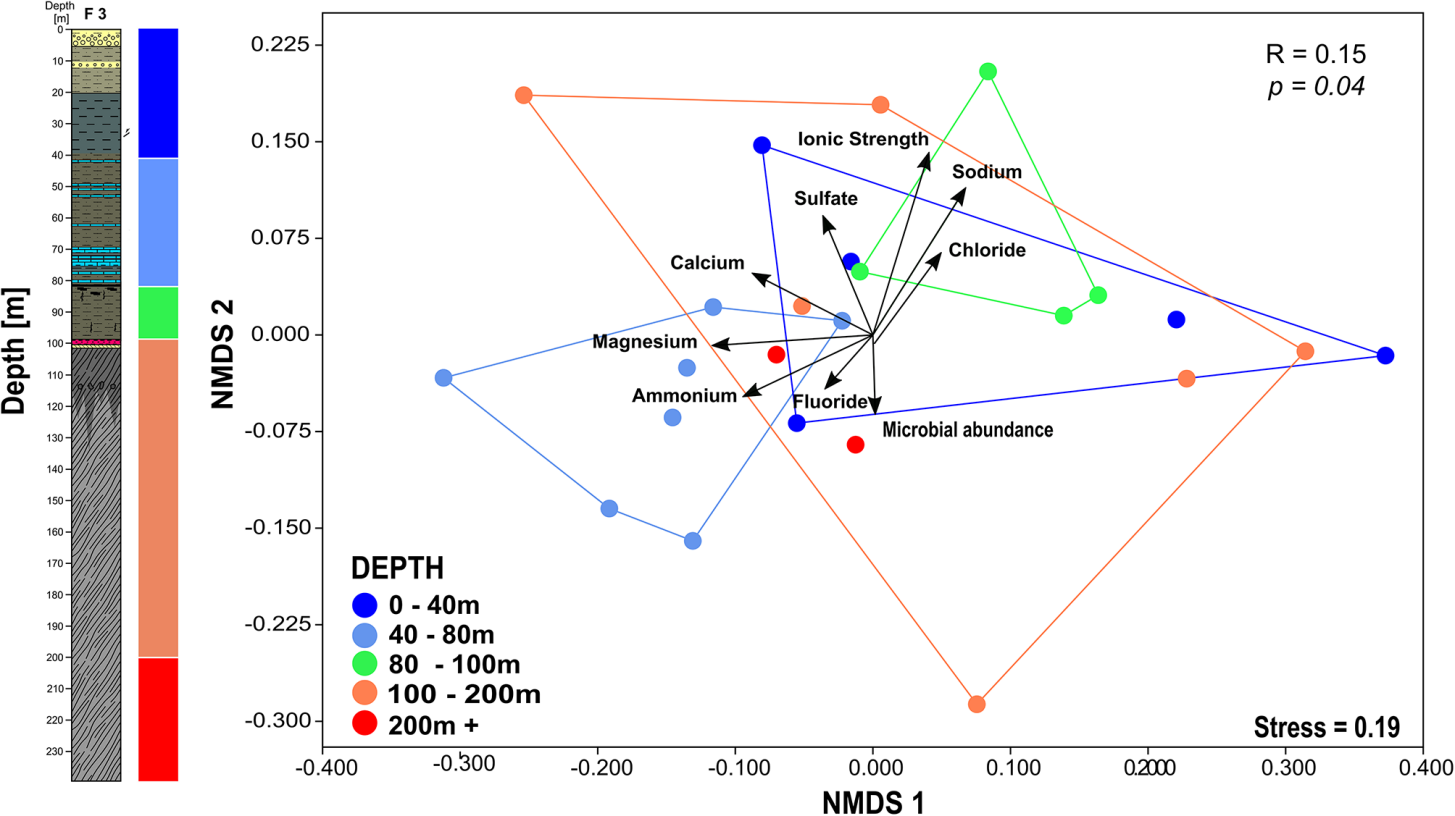
Non-metric dimensional scaling (NMDS) plot displaying microbial diversity across the recovered core samples. Samples are color-coded by depth and have environmental parameters fitted as vectors

Non-metric multidimensional scaling was conducted to evaluate potential differences in microbial community structure across the recovered drill core samples (Fig. 4) and to compare the recovered microbial communities with those identified in the control drill mud samples (Figure S3). Drill mud samples (DM) were found to cluster separately from drill core samples, with only DM1 located in the vicinity of the analyzed sediments (Figure S3).

Clustering by depth was observed for samples shallower than 45 m and between 50 and 100 m (Fig. 4), suggesting a shift in community across these regions. Microbial communities in deeper sediments were found to vary in diversity, as no distinct clusters could be identified. Similarly, samples associated with higher ion concentrations (ionic strength > 1000 mg L^− 1^) were found to cluster close to each other, whereas the greatest diversity was observed among samples characterized by lower ion concentrations (Fig. 4, Figure S4). Differences in community composition between samples grouped by depth or ionic concentration were further assessed using ANOSM (analysis of similarity) and PERMANOVA (permutational multivariate analysis of variance) calculations. These procedures further support the graphical consideration, as differences between communities in shallow sediments (up to 50 m) and intermediate sediments (50–100 m) were found to be significant (ANOSIM, *p* = 0.02) or close to significant (PERMANOVA, *p* = 0.06). Similarly, grouping dependent on ion concentration is also supported by this approach. Samples with high ionic content (> 1000 ppm) were found to be microbiologically different from samples with very low (> 200 ppm) and low (200–500 ppm) ionic content using both ANOSIM (*p* = 0.03 and *p* = 0.00) and PERMANOVA (both *p* = 0.05). Graphical ordination and statistical analysis revealed microbial community composition in Eger sediments to change with depth in the upper and intermediate regions and to differ between sediments with high and low ionic content.

### Correlation and similarity analysis

To connect ecological, physical, and geochemical measurements, we conducted a correlation analysis. Using Spearman rank coefficients, it was possible to identify a strong, significant inverse correlation between sediment depth and the concentrations of ammonium, calcium and magnesium ions (*p* < 0.05) (Figure S5). Finally, we were able to detect a strong, significant correlation between the number of counted cells and depth (*R* = 0.73, *p* < 0.05), however, as cells could only be counted for about half of the samples, we believe this finding should be interpreted with caution.

### Microbial community structure

Evaluation of microbial diversity and composition across the drill core revealed a Bacteria dominated community enriched in soil and water-associated microbes. Bacteria were dominant across all samples, with relative abundances ranging between 90.6% and 100%. Meanwhile, archaea were especially abundant in samples from depths between 30 and 42 m, (3.3–8.3%), 65 m (9.4%), 108 m (2.9%), and in deeper sediments between 185 and 230 m (1.0 − 6.9%). In all other samples, Archaea were detected at relative abundance below 1%.

Generally, three different microbial community composition patterns were observed based on the available 16 S rRNA sequencing data. Down to a depth of 42 m drill core sediments were dominated by Alpha- und Gammaproteobacteria (Fig. 6, Figure S6), as especially the genera *Phyllobacterium* (up to 42.1%), *Sphingomonas* (up to 43.9%), and *Pseudomonas* (up to 41.2%) were frequently detected. Phylogenetic classification of the most abundant *Sphingomonas* ASV (ASV 12) suggested a close relationship to *Sphingomonas echinoides* strain NBRC 15,742 (BLAST 100% identity over 468 bp) and an environmental *Sphingomonas* clone, previously identified in hydrocarbon-contaminated soil (Figure S7). *Phyllobacterium* sequences were most closely associated with *Phyllobacterium bourgognense* strain STM 201 and *Phyllobacterium zundukense* strain Tri-48 (BLAST 100% identify over 468 bp, data not shown). The detected *Pseudomonas* ASVs are related to *Pseudomonas peli* strain R-20,805 (BLAST identify over 469 bp) and also highly similar to uncultured *Pseudomonas* spp. discovered in subsurface water [71] (Figure S7). In addition to these abundant taxa, sediments above 42 m were also characterized by the frequent detection of the genera *Alshewanella* (up to 11.4%), *Caldiscericum* (up to 3.3%), and Chloroflexi of the taxon Dehalococcoida (up to 6.5%). Sediments recovered from the shallowest collected sample (17 m) had a unique microbial signature, as members of the Comamonadacaeae, particularly *Acidovorax* and (12.2%) and *Rhodoferax* (8.4%) were enriched.

**Fig. 5:**
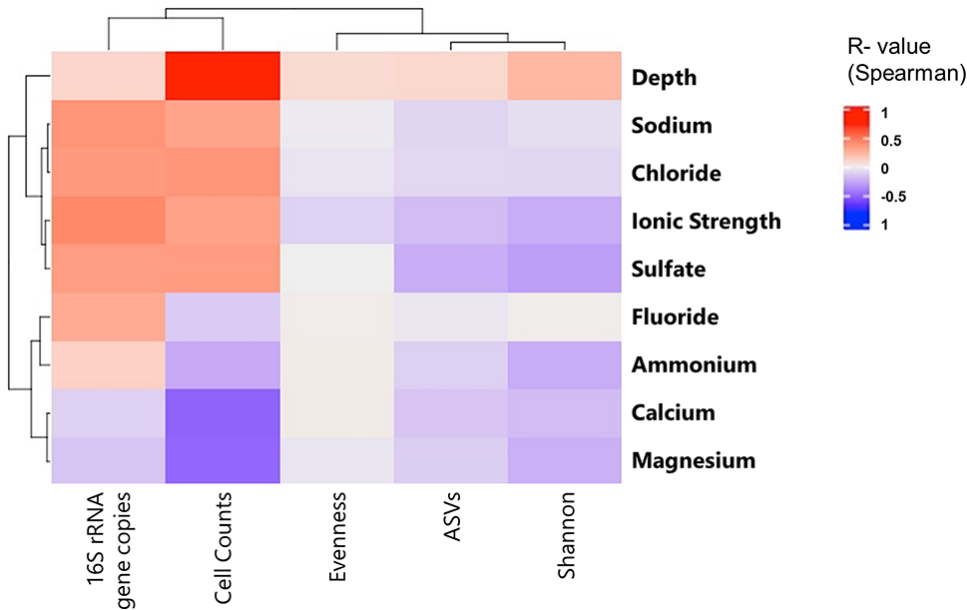
Correlation of microbiological, environmental, and geochemical parameters based on Spearman correlation coefficients

**Fig. 6:**
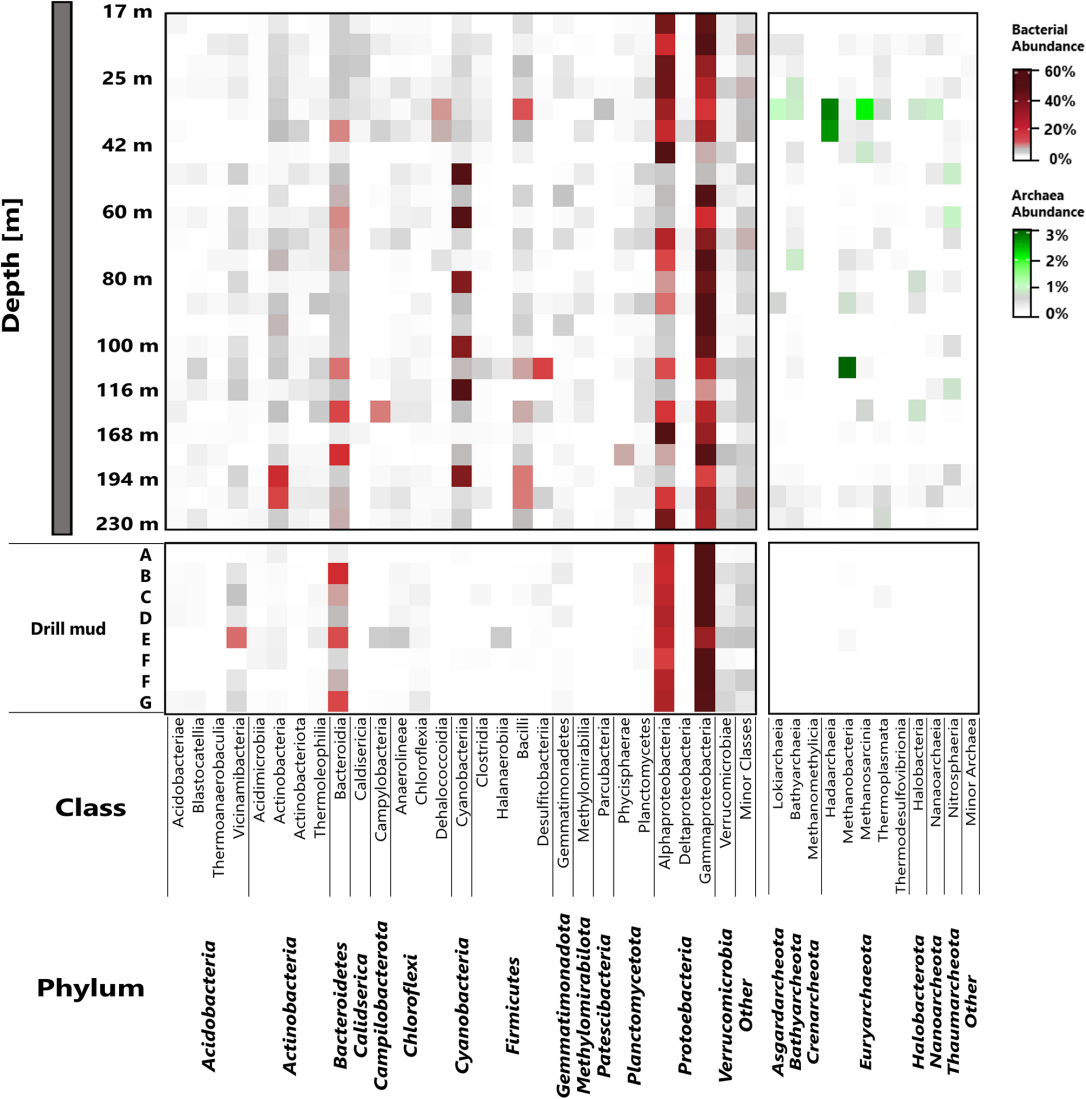
Class level microbial composition for bacteria (red) and archaea (green) across recovered drill core samples and collected drill mud samples

Below 42 m, drill core microbial communities were more variable, as Alphaproteobacteria were less abundant and Gammaproteobacteria, especially *Pseudomonas* and *Alshewanella*, increased in abundance. Particularly notable was the frequent detection of several ASVs closely associated with freshwater Cyanobacteria species, especially *Cyanobium*, *Synechococcus*, and *Snowella*. However, high numbers of Cyanobacteria-affiliated sequences were not detected continuously, but only at certain intervals (Fig. 6, Figure S5, Figure S6). Cyanobacteria abundance was highest in samples from depths of 48 m (69.4%), 60 m (53.3%), and 116 m (66.9%) (Fig. 5, Figure S6). Between 108 m and 136 m sediments were more enriched in sulfur cycle-associated taxa such as *Sulfurimonas* (6.2% at 136 m) and the strict anaerobic, acidophilic *Desulfosporosinus* (15.1% at 108 m), as well as the chemolithoautrophic and halophilic taxon *Thiohalophilus* (4.5% at 135 m). Also notable was the detection of *Roseisolibacter* (albeit at low abundances of up to 3.9% at 54 and 87 m).

Deep sediments (150 m and below) were characterized by lower *Pseudomonas* counts, instead sequences affiliated with Firmicutes (*Bacillus*, 195–220 m) and Actinobacteria (*Streptomyces* 195–230 m) were detected more frequently. Another Cyanobacteria enrichment was identified at a depth of 195 m, as *Cyanobium* (31.2%) and Microcystis (4.1) signatures were enriched (Fig. 6, Figure S6).

**Fig. 7:**
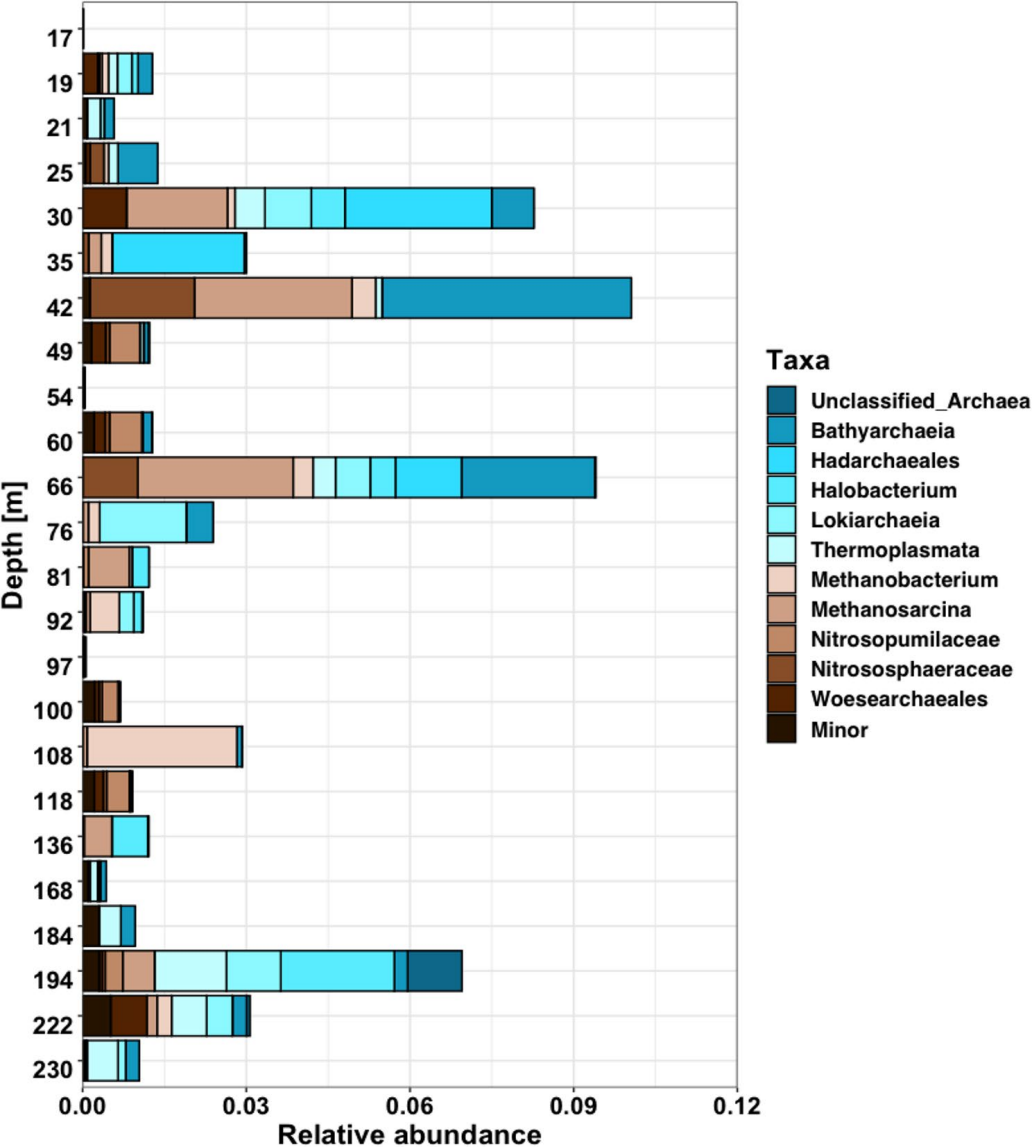
Distribution and relative abundance of archaea across the recovered drill core sediments

One specific focus of this study was the evaluation of the archaeal fraction, as the occurrence of CO_2_ and H_2_ utilizing, methanogenic archaea is highly relevant to this type of degassing environment and may provide support for the microbial utilization of tectonically released substrates, specifically H_2_. Analysis of the archaeal fraction revealed a large diversity, with 173 different archaeal ASVs detected. Our data indicates the presence of Euryarchaeota, Crenarchaeota, and Nanoarchaeota throughout the drill core (Fig. 7). Archaeal signatures were strongest in sediments from a depth of around 30 to 40 m, as Hadarchaeia (up to 2.8%), Bathyarcheia (up to 4.5%), and *Methanosarcina* (up to 2.8%) were especially abundant. *Methanobacteria* sequences were identified in most sediments, but were especially enriched at a depth of 108 m (2.7%). Thermophilic Thermoplasmata were also present in the majority of the evaluated core samples, and were most abundant at a depth of 35 m (0.6%) and in the deepest sediments around 220–230 m (0.4–1.3%). Similar to Thermoplasmata, Lokiarchaeia and Nanoarchaeia were detected at their highest frequencies (up to 0.9%) at these depths.

Methanogenic taxa, especially *Methanosarcina* and *Methanobacteria* were detected frequently and were found to be one of the most abundant archaeal groups, particularly in sediments from intermediate and shallow depths. A closer examination of the abundant *Methanosarcina* ASVs suggested that the observed communities were closely related to *Methanosarcina spelaei* strain MC-15 (100% identity over 254 bp), while the detected *Methanobacteria* ASVs were closely affiliated with *Methanobacterium oryzae* (99.21% identity over 254 bp), *Methanobacterium lacus* (96.04% identify over 254 bp), and unclassified *Methanobacterium* environmental sequences from lake sediments (Tree Figure S7).

Finally, several ASVs belonging to the ammonia-oxidizing and often CO_2_-fixing Nitrososphaerota were detected across the analyzed community (Fig. 7). Nitrosopumilaceae (up to 0.6% at 60 m) and Nitrososphaeraceae (up to 1.9% at 42 m) were the most abundant and frequently occurring groups.

## Discussion

### Unique ecosystem with abundant CO_2_

In this study, we examined the geochemical and microbial composition of subsurface sediments from the Cheb Basin in the Eger Rift, using up to 238 m deep core samples from the Hartoušov Mofette Field. Sedimentological analysis identified three distinct formations in the upper 100 m, including swamp-associated mudstone with root structures and peat/lignite fragments, which were also reflected by TOC and TON measurements from these sediments.

Deeper drill core samples varied between slightly and heavily weathered schist, characteristic of recurrent groundwater movement, and highlighted by the frequently present CO_2_ (CO_2_ bubbling was observed when examining cores). The presence of minerals such as siderite, kaolinite, and gypsum, as well as the partial softening of the mica schists in this section indicate the influence of near-surface weathering processes that followed zones of increased permeability caused by joints, foliations, and faults during the late Mesozoic or early Cenozoic [61]. This heterogenous section can therefore be considered a macrohabitat, with elevated transport through CO_2_ upwelling and groundwater fluctuations, which may contain many different ecological niches.

Despite challenges in DNA recovery due to low biomass and varied core material, 16S rRNA sequencing revealed a unique microbial ecosystem. While common soil and root microbes dominated superficially, deeper analysis suggested distinct microhabitats shaped by geological and geochemical conditions, inhabited by heterotrophic, chemoautotrophic, acidophilic, and methanogenic microorganisms.

### Variable bacteria community dominated by soil and water microorganisms

Evaluation of the microbial community structure across the 230 m-long drill core revealed a low-biomass community dominated by heterotrophic soil and surface water bacteria. Distinct communities were observed in the upper 100 m, while microbial populations in deeper, weathered schist sediments were more heterogeneous, matching findings made previously by Liu et al. [29] across rock formations at depths between 70 m and 95 at the same location.

This interval was particularly enriched in microaerophilic or facultative anaerobic heterotrophs of the genera *Pseudomonas* and *Alshewanella*, previously identified in various subsurface settings such as hydrocarbon environments, drill cores, and deep groundwater [53, 72–74]. Further phylogenetic analysis suggested these taxa are closely related to strains from surface water bodies. Elevated sulfate, sodium, and calcium concentrations in these sediment layers emphasize that this area is geochemically divergent from the surrounding subsurface, possibly driven by CO_2_ accumulation in a subsurface aquifer as suggested by Liu et al. [29] and Bussert et al. [31] or general groundwater movement in this area. The nearby Pleśna River, which may serve as a connection to surface water bodies, supports this hypothesis. As recent analyses of Pleśna River water samples, taken from close to the drill site were enriched in taxa such as *Rhodoferax*, *Pseudomonas*, and Comamonadaceae (data not published).

Below 100 m, microbial communities showed no clear correlation with geochemical or geological parameters, reflecting the heterogeneous nature of the weathered schist. Increased cations and slightly higher biomass at the deepest core levels may indicate another CO_2_-rich aquifer, however, because only a small number of samples from these depths were available for this study, it is not possible to make any detailed statements on the microbial ecology in these regions.

### CO_2_ driven communities are not abundant but persistent

Our study did not reveal a rich microbial community of CO_2_ fixating microorganisms or even a strong abundance of acidophiles, as hypothesized based on the unique CO_2_ degassing conditions. However, taxa with these functional traits were present throughout the samples, indicating the unique environmental feature could have ecological influence. Consistent with prior studies of high CO_2_ environments [11, 75, 76], including the Eger subsurface [29, 44], we frequently identified taxa belonging to the Comamonadaceae family, a diverse group of water and soil bacteria. The here discovered genera *Acidovorax*, *Rhodoferax*, *Hydrogenphaga*, and *Curvibacter* have all been detected in high CO_2_ environments [12, 13, 77, 78], and were specifically observed in a freshwater aquifer after a simulated carbon storage leakage [11, 12]. Certain members of these taxa have been suggested to fix or utilize CO_2_ or H_2_ in their likely mixotrophic lifestyles [11, 79]. Another finding potentially supporting a somewhat CO_2_-driven bacterial community is the frequent detection of the taxa *Desulfosporosinus* and *Sulfurimonas*, both of which have been discovered in high CO_2_ and acidic environments, such as McElmo Dome [11, 13, 80–82]. While members of the *Desulfosporosinus* are moderate acidophilic sulfate reducers with the metabolic capability to fix CO_2_ into acetyl-CoA via the Wood-Ljungdahl pathway [83], *Sulfurimonas* may drive carbon fixation by rTCA cycling, via pathways of sulfur/hydrogen oxidation [84]. Additionally, ongoing work by our group showed that Eger sediment enrichments incubated under a H_2_/CO_2_ atmosphere were highly abundant in *Desulfosporosinus* [42]. Although no correlation was found between this taxon’s occurrence and geochemical parameters like sulfate, our data observations suggest that it thrives under high CO_2_ conditions and likely contributes to sulfur cycling in the Eger subsurface. Like *Sulfurimonas*, members of the single-species genus *Thiohalophilus* are chemoautotrophic sulfur oxidizers. However, these organisms can assimilate CO_2_ via the Calvin–Benson–Bassham cycle [85] and usually occur in hypersaline lakes [86]. Although only occasionally detected in our samples, their presence may indicate a direct and indirect influence of CO_2_ on the Eger subsurface, where acidification could enhance ion dissolution and create saline niches suitable for these microbes.

Other acidophilic taxa, including Acidobacteriales and Acidothermales, were detected at low abundances throughout the Eger core, consistent with prior findings in mofette and groundwater samples from the Cheb Basin [37, 40]. Their co-occurrence with acidophilic sulfur oxidizers, such as *Sulfurimonas* and *Thiohalophilus*, supports the idea of ecological niches driven by CO_2_ and ascending saline groundwater, harboring acidophilic and halophilic microbial populations.

While several of the identified and described taxa matched those detected in other natural McElmo Dome [13] and artificial CO_2_ environments [11, 23], we did not detect any of the CO_2_ adapted candidatus taxa observed in Crystal Geyser samples [22]. While the obvious geographical and environmental differences between the two sites are simple explanations for this, current efforts to obtain genomic material for metagenomic analyses will allow us to conduct a more detailed comparison between these two natural high CO_2_ sites.

### The role of cyanobacteria remains unclear

One of the least anticipated findings of this study was the frequent detection of Cyanobacteria belonging to the phototrophic taxa *Cyanobium*, *Synechococcus*, *Microcystis*, and *Snowella* at specific depth intervals. The presence of Cyanobacteria in subsurface settings is not uncommon. These types of microorganisms have been previously detected in various types of subsurface environments at relatively high abundances [82, 87, 88]. However, the fact that the taxa discovered here are usually associated with surface water bodies and an aerobic, phototrophic lifestyle [89] makes their discovery unexpected. We initially suspected on-site or lab contamination, but multiple sequenced drill mud and wet lab controls showed no abundant Cyanobacteria ASVs. Furthermore, the Cyanobacteria ASVs only occurred at very specific depth intervals, arguing against a general external contamination source. Our observations also indicate these microbial populations to be native, as their presence was found to be statistically correlated with taxa typically found in the subsurface, including Gaiellales [90, 91], Woesearchaeota [92], and in soil and acidic environments, including *Terrimonas* and Acidobacteriales [41, 93, 94].

At this point, we do not have a simple explanation for this phenomenon. However, the distinct pockets of cyanobacterial abundance may be explained by periodic, vertical groundwater movement in the Cheb Basin, which is characterized by mineral springs and proximity to surface water, such as the Pleśna River. Thus, seasonal or periodic washing of Cyanobacteria into the subsurface is a plausible hypothesis, supported by their absence in samples from the 2016 drilling.

Another possibility is that the Cyanobacteria represent ancient DNA from sediment deposition. However, the high abundances we observed make this explanation less likely. Further investigation, such as regular groundwater testing or additional drilling, are needed to explore this phenomenon in more detail.

### Large diversity highlights importance of archaeal communities

One of the most important goals of this study, was to evaluate the presence and diversity of archaea. The Eger subsurface has been hypothesized to provide methanogenic substrates, H_2_ through frequent swarm earthquake activity and CO_2_ via mantle-derived degassing [27, 28, 31], with primary production through methanogenic archaea providing the basis for a secondary heterotrophic lifestyle by bacteria. Low abundances of Euryarchaea have been detected in Cheb Basin subsurface sediments [29] and mofette and spring waters from the region [44]. A recent study [42] also emphasized the ability of methanogenic archaea residing in the Eger subsurface sediments to become active and produce methane when exposed to a H_2_/CO_2_ atmosphere. Hence, it was hypothesized that the seismic release of H_2_ triggers methanogenic activity and that a small, dormant community of methanogenic archaea might become active following such an event. Our observations strongly support the importance of Archaea in the Eger subsurface and provide additional evidence that the terrestrial subsurface is home to a significant diversity of archaeal populations.

Similar to Liu et al. [29], we detected methanogenic Euryarchaeota, especially *Methanobacterium* and *Methanosarcina*, across almost the entire length of the analyzed core. These taxa were most abundant in shallow and intermediate sediments (to 110 m), but their presence in deeper samples suggests persistent methanogenic activity. Additional cultivation work allowed us to grow *Methanobacterium* and *Methanosarcina* enrichments using CO_2_/H_2_ from various Eger sediment samples, even those where these organisms were present at minimal levels [42]. *Methanobacterium* specializes in CO_2_ reduction with H_2_, a strictly hydrogenotrophic process [95–97]. *Methanosarcina*, capable of all three methanogenic pathways, is highly versatile, tolerating acidic, thermophilic, and halophilic conditions. Certain *Methanosarcina* species can also utilize acetate or methylated compounds to form CO_2_, but have also been suggested to produce acetate from carbon monoxide and thus provide the building blocks for secondary metabolisms, which may be essential in a subsurface environment otherwise scarce in organic substrates [8, 96–98].

Although our amplicon data does not allow us to specify strains, the frequent detection of these methanogens supports the hypothesis of a persistent Eger rift methanogenic community. Methanogenesis likely occurs when H_2_ is available, producing biogenic methane as observed by Bräuer et al. [26, 27]. Both *Methanobacterium* and *Methanosarcina* ASVs were found to occur with sulfate and thiosulfate-reducing and fermentative Proteobacteria and Firmicutes, such as *Desulfosporosinus*, *Halanaerobium* and *Bacillus*, highlighting diverse microbial metabolisms. While sulfate reduction typically restricts methanogenesis, both processes have been observed to coexist in sediments and high CO_2_ settings [99–102]. Active methanogenesis has also been observed at the nearby Wettingquelle [103], while methanogens enriched from Eger sediments were actively producing methane, despite the presence of sulfate reducers [42]. Thus, acetolactic, hydrogen-producing, sulfate-reducing bacteria and hydrogenotrophic methanogens coexist, which could be the case in the Eger subsurface.

While the detection of methanogenic archaea provided new insights into methanogenic processes in the Eger rift subsurface, our exploration also highlighted various other Euryarchaeota species. Acidophilic Themoplasmata and halophilic Halobacteria were frequently detected, likely reflecting adaptation to the unique CO_2_ degassing conditions [97, 104]. Another notable finding was the high abundance of Hadarchaeales in selected samples, as this relatively newly established group of *Candidatus* microorganisms is closely associated with both marine and terrestrial subsurface settings and was first discovered in acidic hot springs [8, 105]. Known for versatile metabolisms, including hydrogen and one-carbon compound oxidation, Hadarchaeales highlight the metabolic diversity of the Eger subsurface [84, 106].

Additional archaeal taxa further underscore this diversity. Bathyarchaeota and Nitrosphaera were detected in intermediate-depth sediments (42 m, 66 m), while Lokiarchaeia were present in shallow and deep samples. Bathyarchaeota, a globally distributed phylum found in diverse environments (marine and terrestrial sediments, hot springs, hydrothermal vents) [43, 107–110], may utilize CO_2_ through reductive acetogenesis via an archaeal Wood–Ljungdahl pathway, suggesting that their frequent detection may be associated with the presence of CO_2_ and H_2_ in the Eger subsurface [107, 111].

Nitrosphaera can oxidize ammonia under aerobic or anaerobic conditions and thrive in high-salinity and low-pH (down to 3.5) environments [112–115]. Interestingly, several genera (Nitrosopumilaceae, *Nitrosarchaeum*) were found to occur with bacterial nitrite and ammonia oxidizers, as well Cyanobacteria, suggesting their distribution could be influenced by the surrounding surface water and even agricultural run-off.

Lokiarchaeia (Asgard superphylum) were initially found in ocean sediments but are now known from terrestrial anaerobic habitats, including hot springs and cave systems [116, 117]. These archaea share ancestral links to eukaryotes, but their metabolic capabilities and thus their potential role in the Eger Rift remain largely unknown.

Exploration of archaeal subsurface communities revealed a diverse, well-adapted population shaped by the unique geological and environmental features of the Eger Rift. Methanogenic archaea appear to be key contributors to microbial processes, likely utilizing available resources such as CO_2_ and H_2_ when present. However, these findings are based on 16 S rRNA amplicon sequencing, which limits taxonomic resolution and provides no insights into viability or metabolic capabilities. Recent recovery of nearly complete draft genomes for *Methanobacterium* and *Methanosarcina* [118, 119] offer opportunities for future work. Metagenomic data may help to obtain additional insights into unculturable archaeal groups, so we have intensified our efforts to obtain enough DNA from these difficult, low biomass samples.

### Eger subsurface harbors diverse and adaptable biosphere

Evaluation of the microbial composition in Eger Rift drill core samples revealed a low-biomass yet distinct community shaped by unique geological and environmental features including elevated salinity, CO_2_ degassing, and frequent groundwater movement. While our data does not support the hypothesis of a “microbial hotspot,” it highlights diverse metabolic strategies, including heterotrophic, autotrophic, and chemolithoautotrophic processes. Soil and water associated Proteobacteria, such as *Pseudomonas*, known for their adaptability and metabolic flexibility were observed, alongside diverse archaeal populations, emphasizing their perseverance and importance in CO_2_-rich and terrestrial subsurface environments. Unexpected cyanobacterial signatures at distinct depths suggest groundwater movement influences but warrant further investigation.

Geochemical analyses indicated ion distribution may correlate with microbial composition, as elevated conductivity and certain ions coincided with shifts in community structure. However, most here assessed ions (e.g., chloride, sodium, calcium, magnesium) are likely not the direct product or reactant of the most common types of microbial metabolisms. Therefore, it is unclear whether both ion distribution and the microbial community are driven by the environment, or if the one directly impacts the other. As the dissolution of ions increases salinity and thus creates an environment that could be more favorable for halophilic organisms, we expected to identify correlations between specific taxonomic groups and the abundance of ionic groups, but none of these were observed. It also needs to be noted, that there are additional environmental and geochemical parameters, that may impact the microbial community, which could not be included in this study.

## Conclusion and outlook

The Eger Rift in central Europe features a unique subsurface ecosystem shaped by mantle-derived CO_2_ degassing and intermittent pulses of geogenic hydrogen from swarm earthquakes. This study assessed up to 230 m deep sediments from this distinct habitat, offering novel insights into lithostratigraphy, ionic composition, and particularly microbial diversity. Findings did not support the hypothesis of a microbial hotspot but suggest that the Eger Rift subsurface ecosystem is influenced by groundwater movement, CO_2_ degassing, and the accumulation of CO_2_ in aquifers. Sediments from previously uncharacterized depths below 100 m are highly heterogeneous and harbor various types of microorganisms, with the capability to pursue aerobic, anaerobic, heterotrophic, autotrophic, and chemolithoautotrophic lifestyles. A surprisingly diverse archaeal community strongly supports the presence of methanogenic, autotrophic, and acidophilic archaea, likely utilizing substrates like H_2_ and the ubiquitous CO_2_ released through the region’s volcanic and tectonic activity. At this point, our findings suggest that certain Eger Rift subsurface areas represent frequently changing environments, impacted by varying groundwater levels, run-off from the surrounding surface, and are in potentially regular exchange with surrounding surface water bodies. On-going investigations focusing on greater taxonomic resolution, microbial behavior, and microbial processes via metagenomics, together with data from this study, have the potential to provide a more detailed look at microbial life in the Eger Rift subsurface and help further explore bio-geo interactions in CO_2_ enriched deep biosphere settings.

## Electronic supplementary material

Below is the link to the electronic supplementary material.

Supplementary Material 1

## Data Availability

The dataset(s) supporting the conclusions of this article are available Nucleotide Center for Biotechnology Information (NCBI) under the accession number #PRJEB55581 https://www.ncbi.nlm.nih.gov/bioproject/PRJEB55581/.
