# Supplementary material for: Microbial diversity and biogeochemical interactions in the seismically active and CO2- rich Eger Rift ecosystem
Source: Environ Microbiome. 2024 Dec 25;19:113. doi: 10.1186/s40793-024-00651-9 (PMC11669242; doi:10.1186/s40793-024-00651-9)
Supplement: Supplementary file 1 — Supplementary Material 1 [file 40793_2024_651_MOESM1_ESM.docx]

**Microbial Diversity and Biogeochemical Interactions in the Seismically Active and CO_2_- rich Eger Rift Ecosystem**

Daniel Lipus^1^, Zeyu Jia^1^, Megan Sondermann^1^, Robert Bussert^2^, Alexander Bartholomäus^1^, Sizhong Yang^1^, Dirk Wagner^1,3^, Jens Kallmeyer^1^

^1^ GFZ German Research Centre for Geosciences, Section Geomicrobiology, Potsdam, Germany

^2^ Section Applied Geochemistry, Institute of Applied Geosciences, Technische Universität Berlin, Berlin, Germany

^3^ University of Potsdam, Institute of Geosciences, Potsdam, Germany

Daniel Lipus – [dlipus@gfz-potsdam.de](mailto:dlipus@gfz-potsdam.de)

Zeyu Jia – [zjia@gfz-potsdam.de](mailto:zjia@gfz-potsdam.de)

Megan Sondermann - [megan.sondermann@student.uni-luebeck.de](mailto:megan.sondermann@student.uni-luebeck.de)

Robert Bussert - [r.bussert@tu-berlin.de](mailto:r.bussert@tu-berlin.de)

Alexander Bartholomäus – [abartho@gfz-potsdam.de](mailto:abartho@gfz-potsdam.de)

Sizhong Yang – [Syang@gfz-potsdam.de](mailto:Syang@gfz-potsdam.de)

Dirk Wagner – [dwagner@gfz-potsdam.de](mailto:dwagner@gfz-potsdam.de)

Jens Kallmeyer – [kallm@gfz-potsdam.de](mailto:kallm@gfz-potsdam.de)

| **Table S1:** Summary of collected Eger drill core samples | | | | | | |
| --- | --- | --- | --- | --- | --- | --- |
| **Sample**  **ID** | **Depth**  **[m]** | **GEOCHEM,**  **CO_2_ flushed,**  **4^o^C** | **MBIO,**  **Liquid N_2_ flash freeze** | **Drill mud sample Seq** | **Cont. Control**  **(Lab)** | **Comment** |
| 17 | 17.8 | X | X |  | X | In plastic liner |
| 19 | 19.8 | X | X |  | X | In plastic liner |
| 21 | 21.8 | X | X |  |  | In plastic liner |
| 25 | 25.8 | X | X |  | X | In plastic liner |
| 30 | 30.7 | X | X |  | X | In plastic liner |
| 35 | 35.5 | X | X |  | X | In plastic liner |
| 42 | 42.2 | X | X | X | X | In plastic liner |
| 44 | 48.6 | X | X |  |  |  |
| 46 | 54 | X | X |  | X |  |
| 48 | 60.1 | X | X |  |  |  |
| 50 | 66.4 | X | X | X | X |  |
| 52 | 71.1 |  |  |  |  | Fractures and loose material, no MBIO sample, GEOCHEM sample broke |
| 54 | 76.1 | X | X | X | X |  |
| 56 | 80.7 | X | X |  | X |  |
| 58 | 88.4 | X |  |  |  | Sample compromised |
| 59 | 92.1 | X | X |  | X |  |
| 61 | 97 | X | X |  |  |  |
| 62 | 100 | X | X | X | X |  |
| 65 | 118.9 | X | X |  | X |  |
| 68 | 135.9 | X | X |  |  |  |
| 74 | 136.1 | X | X |  |  |  |
| 80 | 154 | X | X | X |  | No PCR product |
| 86 | 168.1 | X | X | X | X |  |
| 93 | 184.2 | X | X | X |  |  |
| 98 | 194 | X | X |  | X |  |
| 99 | 195.2 | X | X |  | X | Contaminated |
| 112 | 222.3 | X | X | X | X |  |
| 116 | 230.5 | X | X |  | X |  |

| **Table S2A:** Geological and geochemical (mg/L) features of the recovered Eger drill core sediment samples | | | | | | | | | | |
| --- | --- | --- | --- | --- | --- | --- | --- | --- | --- | --- |
| **Sample ID** | **Depth [m]** | **Formation** | **Ionic Strength*** | **Sodium** | **Ammonium** | **Magnesium** | **Calcium** | **Fluoride** | **Chloride** | **Sulfate** |
|  |  |  | **µS** | **mg/L** | | | | | | |
| 17 | 17.8 | Quaternary Vildsteijn Fm. | 241 | 7.72 | 57.86 | 19.96 | 147.99 | 0.00 | 0.71 | 212.34 |
| 19 | 19.8 | Quaternary Vildsteijn Fm. | 208 | 6.84 | 63.55 | 16.02 | 111.67 | 0.00 | 0.41 | 185.80 |
| 21 | 21.8 | Cypris Fm | 321 | 7.89 | 67.89 | 29.62 | 215.61 | 0.00 | 0.48 | 303.98 |
| 25 | 25.8 | Cypris Fm | 263 | 18.97 | 69.15 | 18.06 | 145.95 | 0.32 | 0.90 | 233.68 |
| 30 | 30.7 | Cypris Fm | 98 | 41.33 | 14.16 | 0.89 | 6.65 | 2.34 | 5.88 | 54.02 |
| 35 | 35.5 | Cypris Fm | 652 | 210.09 | 113.29 | 27.22 | 210.89 | 0.00 | 7.99 | 533.05 |
| 42 | 42.4 | Cypris Fm | 316 | 132.21 | 26.19 | 4.27 | 25.73 | 0.44 | 10.79 | 103.76 |
| 44 | 48.6 | Cypris Fm | 455 | 187.36 | 28.11 | 5.31 | 50.53 | 0.34 | 15.28 | 161.16 |
| 46 | 54 | Cypris Fm | 515 | 191.10 | 28.12 | 8.24 | 84.43 | 0.18 | 11.43 | 218.79 |
| 48 | 60.1 | Cypris Fm | 383 | 155.73 | 40.05 | 3.67 | 33.40 | 0.34 | 9.94 | 131.84 |
| 50 | 66.4 | Cypris Fm | 295 | 139.18 | 27.11 | 2.09 | 17.47 | 0.45 | 8.02 | 83.91 |
| 54 | 76.1 | Cypris Fm | 1726 | 555.22 | 58.70 | 31.19 | 574.99 | 0.00 | 48.22 | 878.36 |
| 56 | 80.7 | Main Seam Fm | 1394 | 653.62 | 39.79 | 0.00 | 0.00 | 0.95 | 176.42 | 400.41 |
| 59 | 92.1 | Main Seam Fm | 1399 | 577.28 | 26.62 | 0.00 | 0.00 | 0.72 | 153.52 | 385.13 |
| 61 | 97 | Main Seam Fm | 1475 | 606.99 | 30.98 | 0.00 | 0.00 | 0.73 | 159.75 | 397.83 |
| 62 | 100 | Phyllitic mica schist | 892 | 400.04 | 26.95 | 1.39 | 0.00 | 0.66 | 66.93 | 191.17 |
| 65 | 108 | Phyllitic mica schist | 329 | 102.88 | 6.32 | 0.00 | 0.00 | 0.20 | 7.13 | 85.96 |
| 68 | 118 | Phyllitic mica schist | 228 | 90.78 | 5.49 | 0.00 | 0.00 | 0.00 | 4.44 | 129.81 |
| 74 | 135.9 | Phyllitic mica schist | 184 | 149.08 | 7.38 | 0.00 | 0.00 | 0.22 | 8.59 | 48.01 |
| 80 | 164 | Phyllitic mica schist |  | 138.69 | 7.51 | 0.00 | 0.00 | 0.00 | 6.68 | 104.39 |
| 86 | 168.1 | Phyllitic mica schist | 282 | 114.56 | 15.27 | 0.00 | 0.00 | 0.17 | 5.45 | 105.37 |
| 93 | 184.2 | Phyllitic mica schist | 169 | 77.04 | 11.22 | 0.00 | 0.00 | 0.24 | 7.38 | 58.09 |
| 98 | 194 | Phyllitic mica schist | 346 | 46.98 | 13.66 | 0.00 | 0.00 | 0.15 | 4.71 | 45.04 |
| 112 | 222.3 | Phyllitic mica schist | 630 | 311.20 | 25.90 | 0.00 | 0.00 | 0.25 | 42.19 | 238.22 |
| 116 | 230.5 | Phyllitic mica schist | 970 | 473.46 | 33.45 | 0.00 | 0.00 | 0.36 | 84.43 | 282.02 |
|  |  |  |  |  |  |  |  |  |  |  |
| *As assessed with Probe | |  |  |  |  |  |  |  |  |  |

| **Table S2B:** Geological and geochemical (millimolar) features of the recovered Eger drill core sediment samples | | | | | | | | | | |
| --- | --- | --- | --- | --- | --- | --- | --- | --- | --- | --- |
| **Sample ID** | **Depth [m]** | **Formation** | **Ionic Strength*** | **Sodium** | **Ammonium** | **Magnesium** | **Calcium** | **Fluoride** | **Chloride** | **Sulfate** |
|  |  |  | **µS** | **mM** | | | | | | |
| 17 | 17.8 | Quaternary Vildsteijn Fm. | 241 | 0.34 | 3.21 | 0.82 | 3.69 | 0.00 | 0.02 | 2.21 |
| 19 | 19.8 | Quaternary Vildsteijn Fm. | 208 | 0.30 | 3.52 | 0.66 | 2.79 | 0.00 | 0.01 | 1.93 |
| 21 | 21.8 | Cypris Fm | 321 | 0.34 | 3.76 | 1.22 | 5.38 | 0.00 | 0.01 | 3.16 |
| 25 | 25.8 | Cypris Fm | 263 | 0.83 | 3.83 | 0.74 | 3.64 | 0.01 | 0.03 | 2.43 |
| 30 | 30.7 | Cypris Fm | 98 | 1.80 | 0.78 | 0.04 | 0.17 | 0.06 | 0.17 | 0.56 |
| 35 | 35.5 | Cypris Fm | 652 | 9.14 | 6.28 | 1.12 | 5.26 | 0.00 | 0.23 | 5.55 |
| 42 | 42.4 | Cypris Fm | 316 | 5.75 | 1.45 | 0.18 | 0.64 | 0.01 | 0.30 | 1.08 |
| 44 | 48.6 | Cypris Fm | 455 | 8.15 | 1.56 | 0.22 | 1.26 | 0.01 | 0.43 | 1.68 |
| 46 | 54 | Cypris Fm | 515 | 8.31 | 1.56 | 0.34 | 2.11 | 0.00 | 0.32 | 2.28 |
| 48 | 60.1 | Cypris Fm | 383 | 6.77 | 2.22 | 0.15 | 0.83 | 0.01 | 0.28 | 1.37 |
| 50 | 66.4 | Cypris Fm | 295 | 6.05 | 1.50 | 0.09 | 0.44 | 0.01 | 0.23 | 0.87 |
| 54 | 76.1 | Cypris Fm | 1726 | 24.15 | 3.25 | 1.28 | 14.35 | 0.00 | 1.36 | 9.14 |
| 56 | 80.7 | Main Seam Fm | 1394 | 28.43 | 2.21 | 0.00 | 0.00 | 0.02 | 4.98 | 4.17 |
| 59 | 92.1 | Main Seam Fm | 1399 | 25.11 | 1.48 | 0.00 | 0.00 | 0.02 | 4.33 | 4.01 |
| 61 | 97 | Main Seam Fm | 1475 | 26.40 | 1.72 | 0.00 | 0.00 | 0.02 | 4.51 | 4.14 |
| 62 | 100 | Phyllitic mica schist | 892 | 17.40 | 1.49 | 0.06 | 0.00 | 0.02 | 1.89 | 1.99 |
| 65 | 108 | Phyllitic mica schist | 329 | 4.47 | 0.35 | 0.00 | 0.00 | 0.00 | 0.20 | 0.89 |
| 68 | 118 | Phyllitic mica schist | 228 | 3.95 | 0.30 | 0.00 | 0.00 | 0.00 | 0.13 | 1.35 |
| 74 | 135.9 | Phyllitic mica schist | 184 | 6.48 | 0.41 | 0.00 | 0.00 | 0.01 | 0.24 | 0.50 |
| 80 | 164 | Phyllitic mica schist |  | 6.03 | 0.42 | 0.00 | 0.00 | 0.00 | 0.19 | 1.09 |
| 86 | 168.1 | Phyllitic mica schist | 282 | 4.98 | 0.85 | 0.00 | 0.00 | 0.00 | 0.15 | 1.10 |
| 93 | 184.2 | Phyllitic mica schist | 169 | 3.35 | 0.62 | 0.00 | 0.00 | 0.01 | 0.21 | 0.60 |
| 98 | 194 | Phyllitic mica schist | 346 | 2.04 | 0.76 | 0.00 | 0.00 | 0.00 | 0.13 | 0.47 |
| 112 | 222.3 | Phyllitic mica schist | 630 | 13.54 | 1.44 | 0.00 | 0.00 | 0.01 | 1.19 | 2.48 |
| 116 | 230.5 | Phyllitic mica schist | 970 | 20.59 | 1.85 | 0.00 | 0.00 | 0.01 | 2.38 | 2.94 |
|  |  |  |  |  |  |  |  |  |  |  |
| *As assessed with Probe | |  |  |  |  |  |  |  |  |  |

| **Table S3:** Microbiological features of recovered and analyzed Eger drill core sediments | | | | | | | |
| --- | --- | --- | --- | --- | --- | --- | --- |
| **Sample ID** | **Depth [m]** | **Generated 16S rRNA sequences*** | **qPCR** | **Cell counts** | **# ASVs*** | **Shannon*** | **Evenness*** |
|  |  |  | **16S rRNA gene copies per gram** | **Cells per gram** | **rarified to 3473 sequences** | | |
| 17 | 17.8 | 171008 | 4.89E+05 | N/A | 185 | 2.97 | 0.11 |
| 19 | 19.8 | 44430 | 1.07E+04 | 6.14E+02 | 220 | 3.20 | 0.21 |
| 21 | 21.8 | 23870 | 1.30E+04 | N/A | 135 | 3.07 | 0.20 |
| 25 | 25.8 | 45415 | 9.63E+03 | 1.23E+03 | 318 | 4.12 | 0.24 |
| 30 | 30.7 | 9815 | N/A | N/A | 109 | 3.38 | 0.34 |
| 35 | 35.5 | 10549 | 4.89E+05 | N/A | 101 | 3.91 | 0.50 |
| 42 | 42.4 | 33042 | 1.07E+04 | 1.23E+03 | 71 | 1.60 | 0.07 |
| 44 | 48.6 | 73047 | 1.35E+04 | 2.79E+00 | 415 | 4.43 | 0.20 |
| 46 | 54 | 89002 | 9.63E+03 | 1.08E+03 | 253 | 2.91 | 0.07 |
| 48 | 60.1 | 38256 | 3.31E+04 | 1.09E+03 | 406 | 4.35 | 0.19 |
| 50 | 66.4 | 26448 | 3.41E+06 | 1.54E+02 | 194 | 4.02 | 0.29 |
| 54 | 76.1 | 10692 | 4.22E+04 | 3.89E+03 | 100 | 3.07 | 0.24 |
| 56 | 80.7 | 3702 | 2.83E+06 | N/A | 140 | 3.58 | 0.26 |
| 59 | 92.1 | 664882 | 2.61E+06 | N/A | 174 | 3.34 | 0.17 |
| 61 | 97 | 79349 | 1.23E+06 | N/A | 182 | 2.37 | 0.07 |
| 62 | 100 | 52352 | 1.06E+04 | N/A | 276 | 3.28 | 0.12 |
| 65 | 108 | 5102 | 9.30E+03 | 3.38E+03 | 113 | 3.76 | 0.41 |
| 68 | 118 | 113156 | N/A | N/A | 576 | 4.64 | 0.18 |
| 74 | 135.9 | 42524 | 7.67E+02 | N/A | 342 | 4.31 | 0.22 |
| 86 | 168.1 | 38711 | 8.54E+03 | N/A | 144 | 2.09 | 0.06 |
| 93 | 184.2 | 14641 | 1.33E+05 | 1.18E+04 | 158 | 4.11 | 0.39 |
| 98 | 194 | 62632 | 5.72E+05 | N/A | 210 | 3.64 | 0.18 |
| 112 | 222.3 | 25142 | 6.75E+06 | 1.49E+04 | 217 | 4.09 | 0.38 |
| 116 | 230.5 | 49697 | 1.40E+04 | 2.21E+05 | 171 | 3.43 | 0.25 |
| * average from up to three replicates | | |  |  |  |  |  |


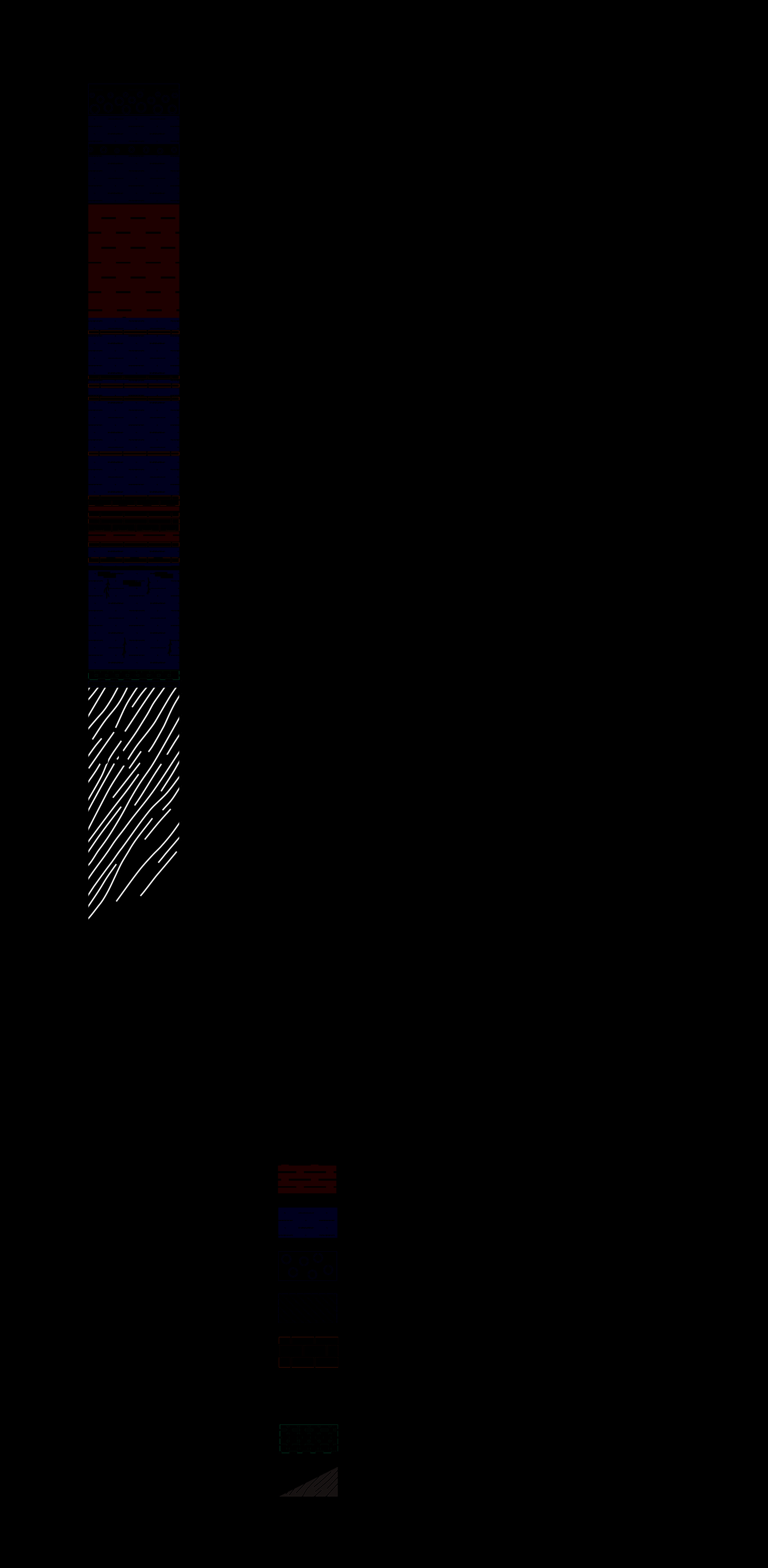


**Figure S1A:** Core profile and detailed description of recovered Eger Rift drill core sediments.

**Figure S1B:** PCA plot based on ionic composition of recovered from Eger Rift sediments and color coded by formations described in Figure S1.


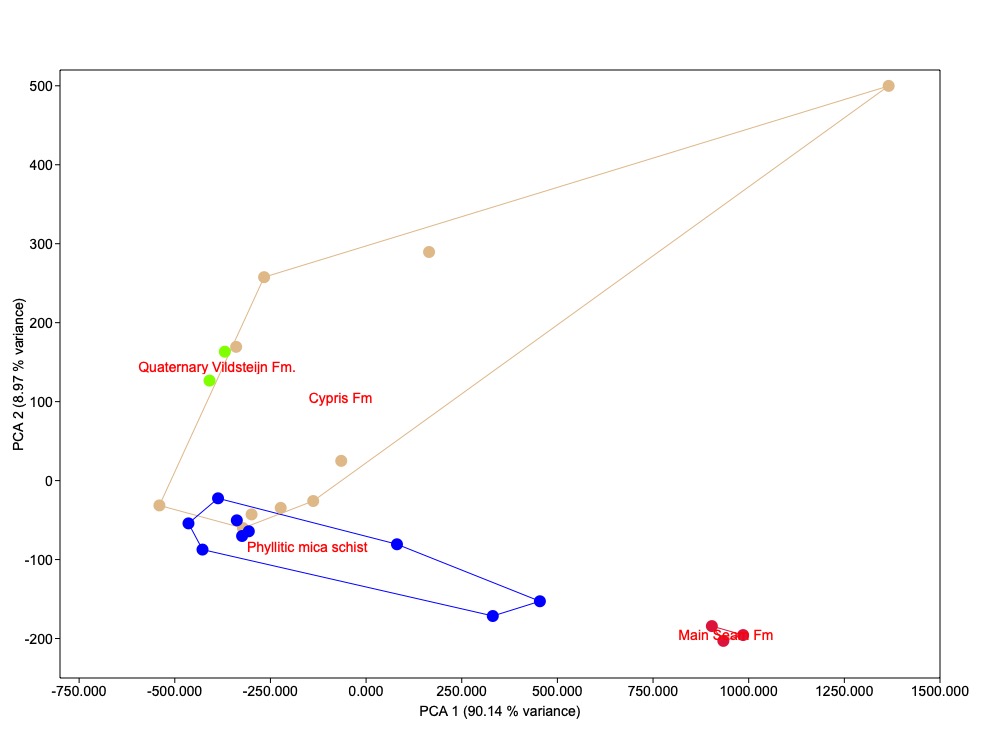


**Figure S2:** PCA plot based on ionic composition of recovered from Eger Rift sediments and color coded by formations described in Figure S1.


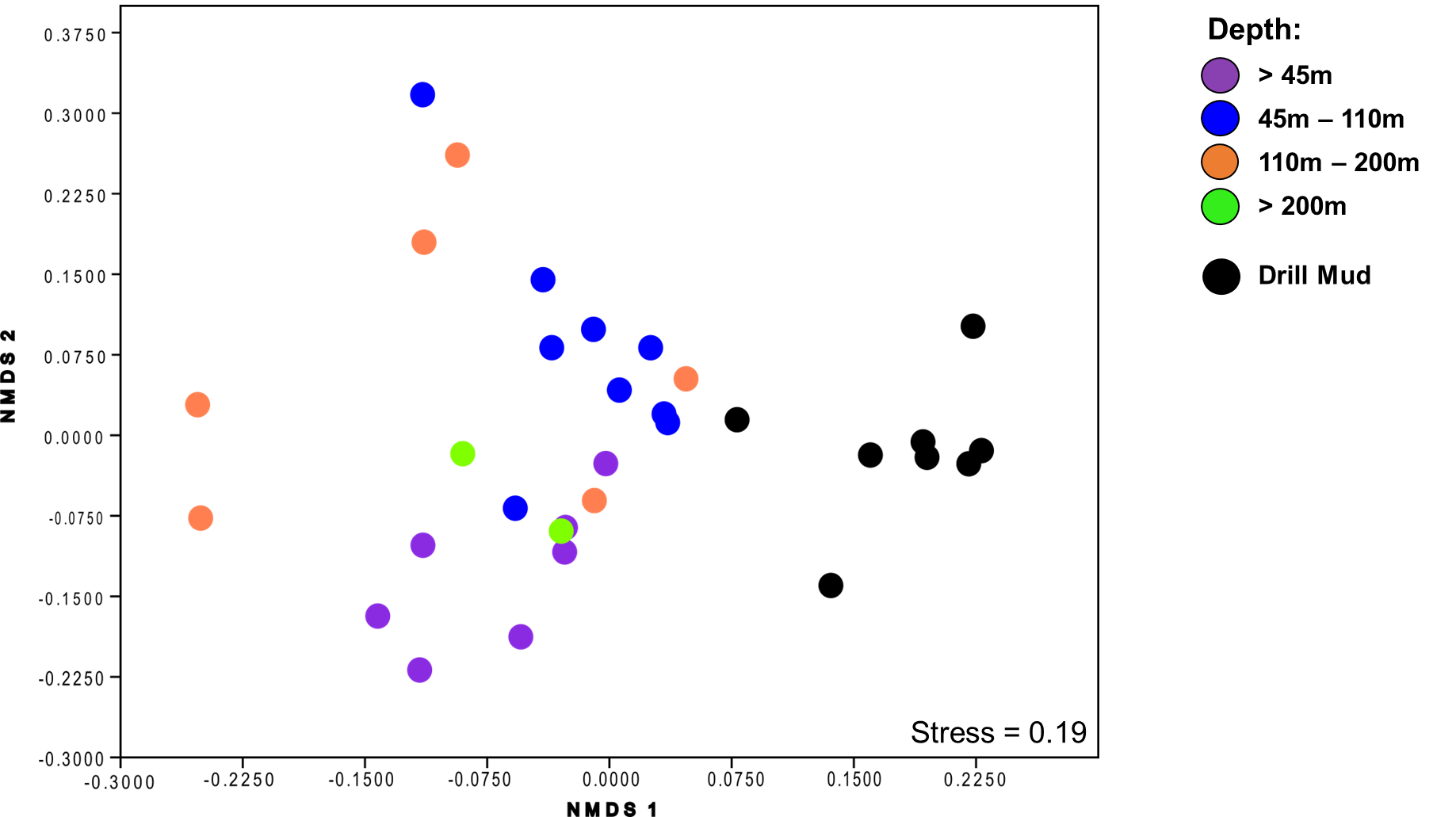


**A)**

**B)**


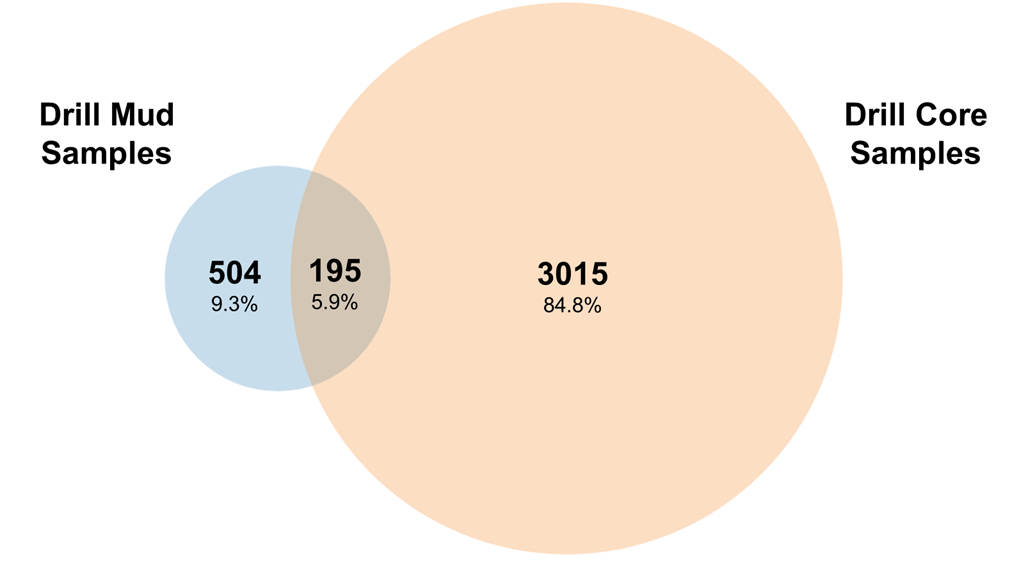


**Figure S3:** (A) Nonmetric multidimensional scaling (NMDS) plot based on microbial community composition, depicting differences in microbial community structure across different drill core depths (color coded) and the analyzed drill core samples. (B) Shared ASVs (relative abundance more than 0.01%) between drill mud and Drill core samples.


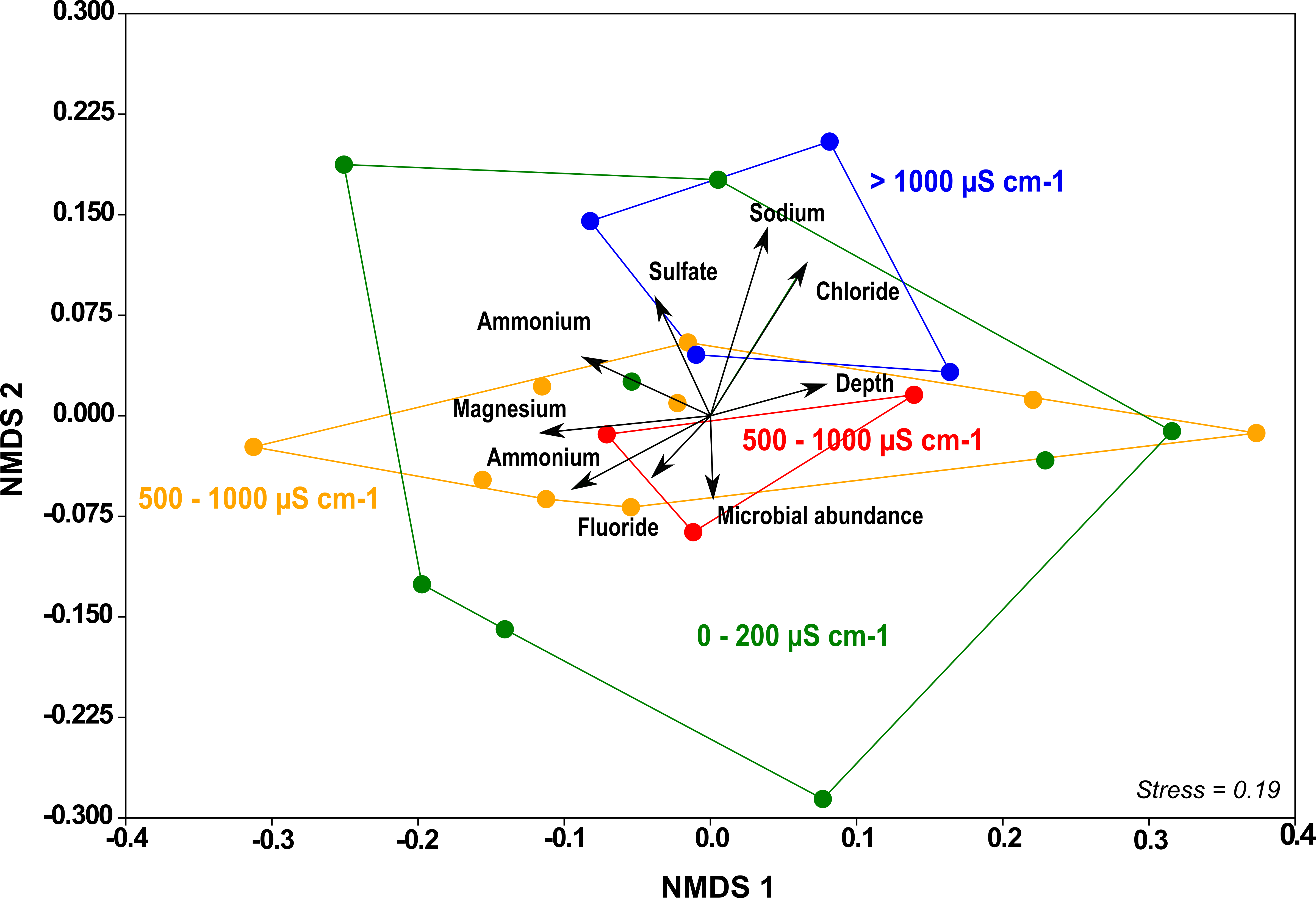


**Figure S4:** Nonmetric multidimensional scaling (NMDS) plot based on microbial community composition, depicting differences in microbial community structure across different ionic concentrations (color coded).


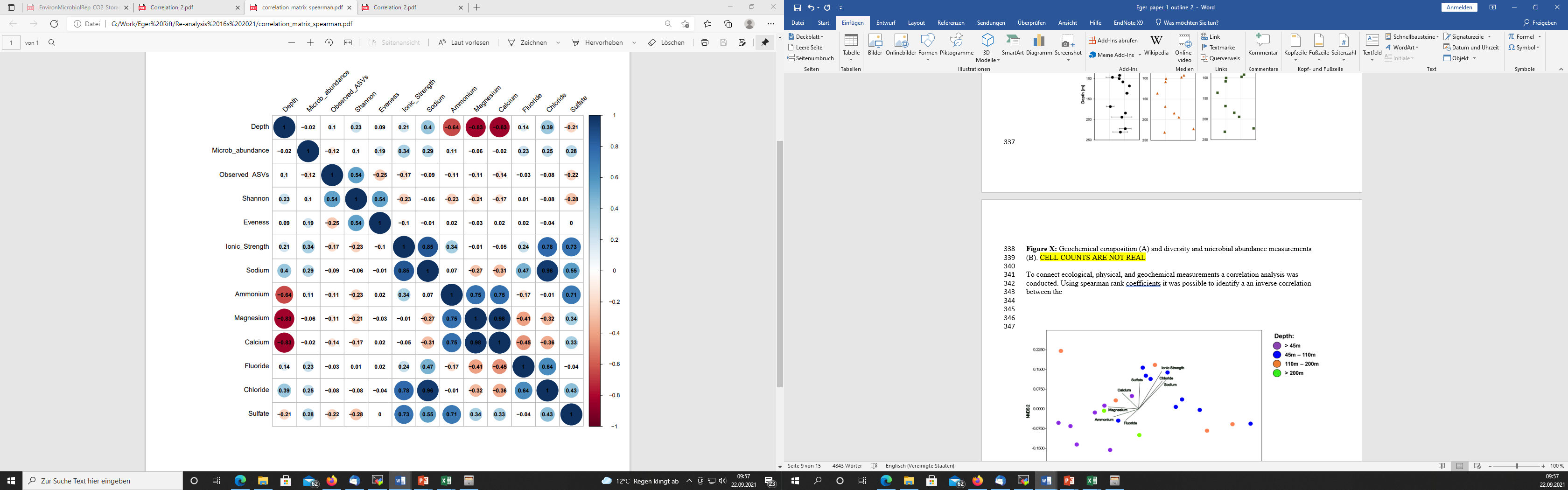


**Figure S5:** Heatmap showing spearman correlation coefficients between microbiological and geochemical measurements.


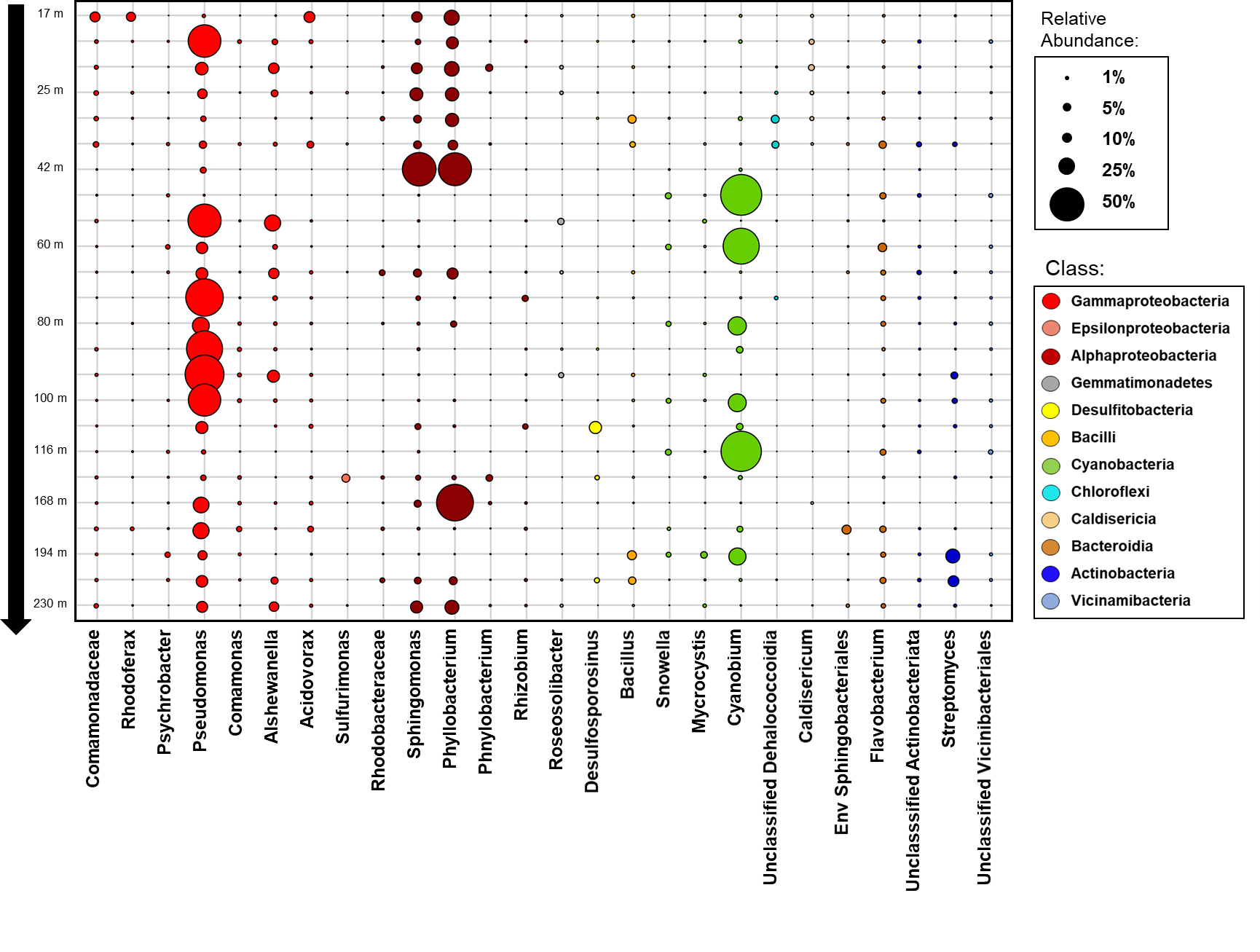


**Figure S6:** Distribution of the 25 most abundant microbial genera identified across the recovered drill core samples, color coded by Class.


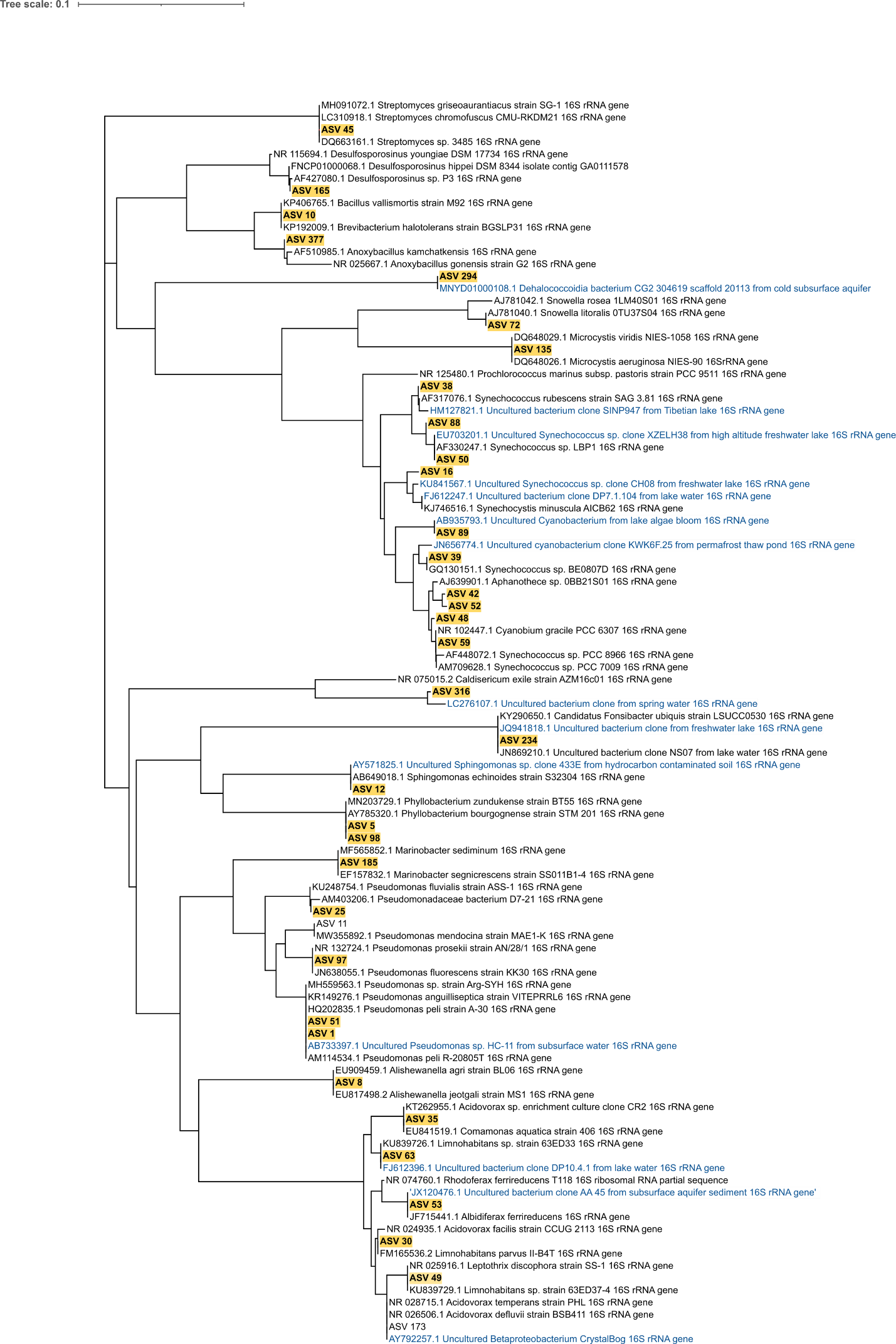

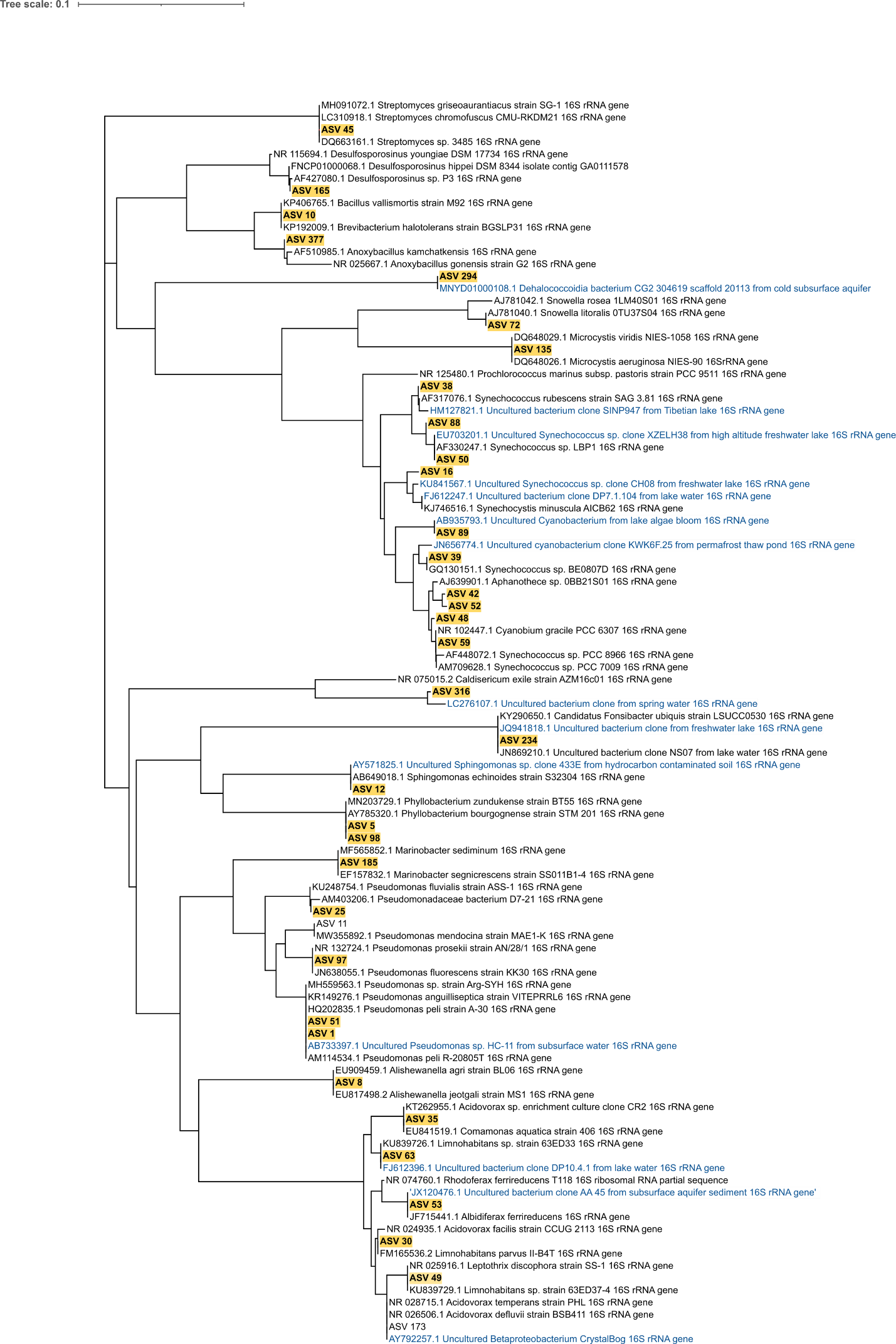


**Figure S7:** 16S rRNA Neighbor joining tree depicting the relationship between the Top 30 identified bacterial ASVs and their closest cultivated neighbors (black) and uncultivated sequences (blue).


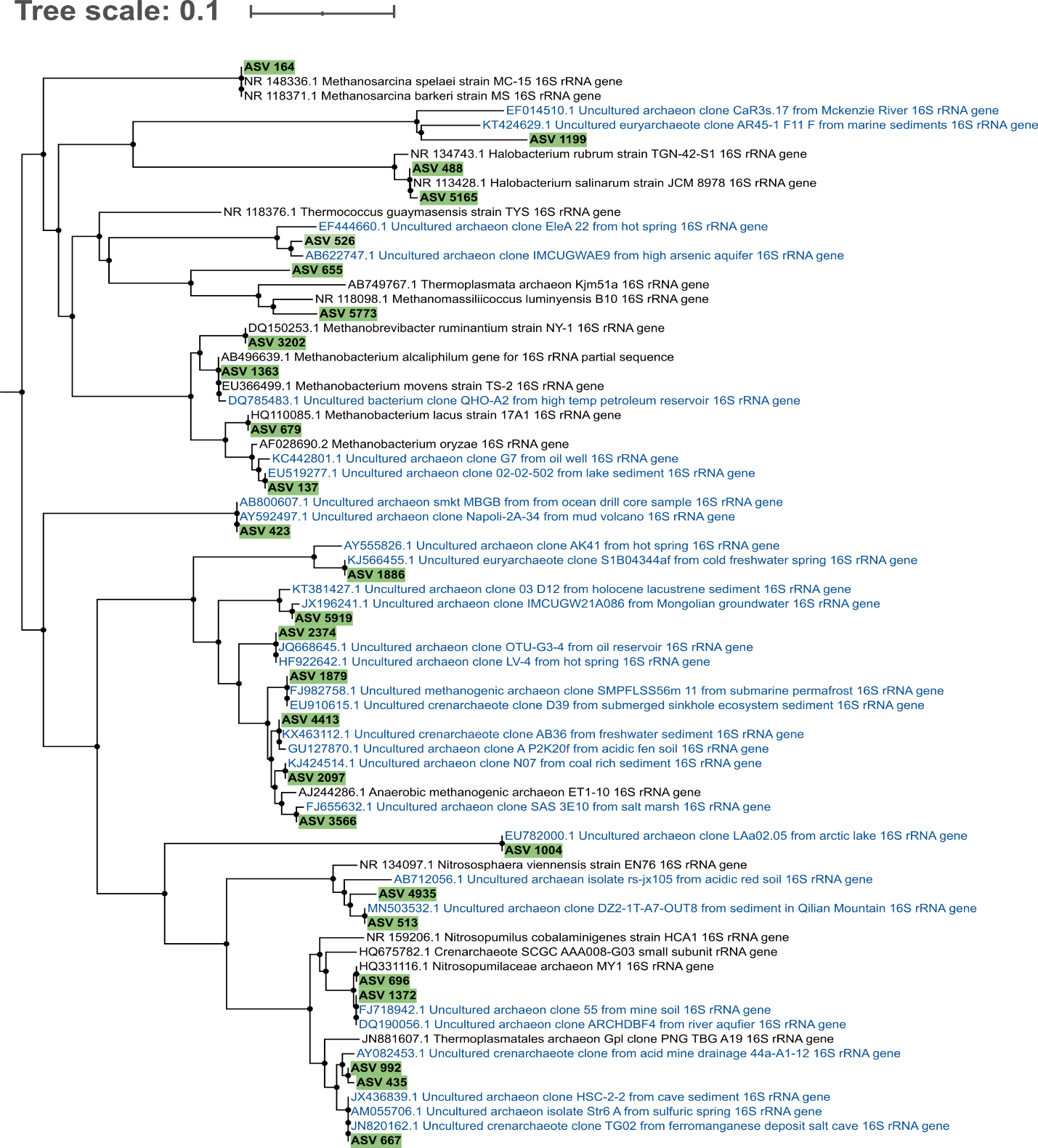


**Figure S8:** 16S rRNA Neighbor joining tree depicting the relationship between the Top 30 identified archaeal ASVs and their closest cultivated neighbors (black) and uncultivated sequences (blue).
